# The Molecular Basis for Apolipoprotein E4 as the Major Risk Factor for Late-Onset Alzheimer's Disease

**DOI:** 10.1016/j.jmb.2019.04.019

**Published:** 2019-05-31

**Authors:** Ana-Caroline Raulin, Lucas Kraft, Youssra K. Al-Hilaly, Wei-Feng Xue, John E. McGeehan, John R. Atack, Louise Serpell

**Affiliations:** 1 Sussex Neuroscience, School of Life Sciences, University of Sussex, Falmer, Brighton, East Sussex BN1 6NN, UK; 2 Sussex Drug Discovery Centre, School of Life Sciences, University of Sussex, Falmer, Brighton, East Sussex , BN1 6NN, UK; 3 School of Biosciences, University of Kent, Canterbury, England CT2 7NJ, UK; 4 School of Biological Sciences, Institute of Biological and Biomedical Sciences, Faculty of Science, University of Portsmouth, Portsmouth, Hampshire PO1 2DY, UK; 5 Chemistry Department, College of Science, Mustansiriyah University, Baghdad, Iraq

**Keywords:** AD, Alzheimer's disease, ApoE, apolipoproteinE, TEM, transmission electron microscopy, SAXS, small-angle x-ray scattering, AUC, analytical ultracentrifugation, CD, circular dichroism, SEC, size exclusion chromatography, MALS, multi-angle light scattering, PB, phosphate buffer, SDS, sodium dodecylsulfate, GuHCl, guanidine hydrochloride, ThT, thioflavin T, apolipoprotein E, Alzheimer's disease, small-angle x-ray scattering, analytical ultracentrifugation, alpha-helix

## Abstract

Apolipoprotein E4 (ApoE4) is one of three (E2, E3 and E4) human isoforms of an α-helical, 299-amino-acid protein. Homozygosity for the ε4 allele is the major genetic risk factor for developing late-onset Alzheimer's disease (AD). ApoE2, ApoE3 and ApoE4 differ at amino acid positions 112 and 158, and these sequence variations may confer conformational differences that underlie their participation in the risk of developing AD. Here, we compared the shape, oligomerization state, conformation and stability of ApoE isoforms using a range of complementary biophysical methods including small-angle x-ray scattering, analytical ultracentrifugation, circular dichroism, x-ray fiber diffraction and transmission electron microscopy We provide an in-depth and definitive study demonstrating that all three proteins are similar in stability and conformation. However, we show that ApoE4 has a propensity to polymerize to form wavy filaments, which do *not* share the characteristics of cross-β amyloid fibrils. Moreover, we provide evidence for the inhibition of ApoE4 fibril formation by ApoE3. This study shows that recombinant ApoE isoforms show no significant differences at the structural or conformational level. However, self-assembly of the ApoE4 isoform may play a role in pathogenesis, and these results open opportunities for uncovering new triggers for AD onset.

## Highlights

• There are three apolipoprotein E isoforms, and E4E4 increases the risk for Alzheimer's disease.
• ApoE2, E3 and E4 share a quaternary, tertiary and secondary structures, and stability.
• ApoE4 forms non-amyloid-like filaments, while ApoE2 and E3 do not.
• Assembly of E4 may play a role increased risk of late-onset Alzheimer's disease.

## Graphical abstract

**Figure:**
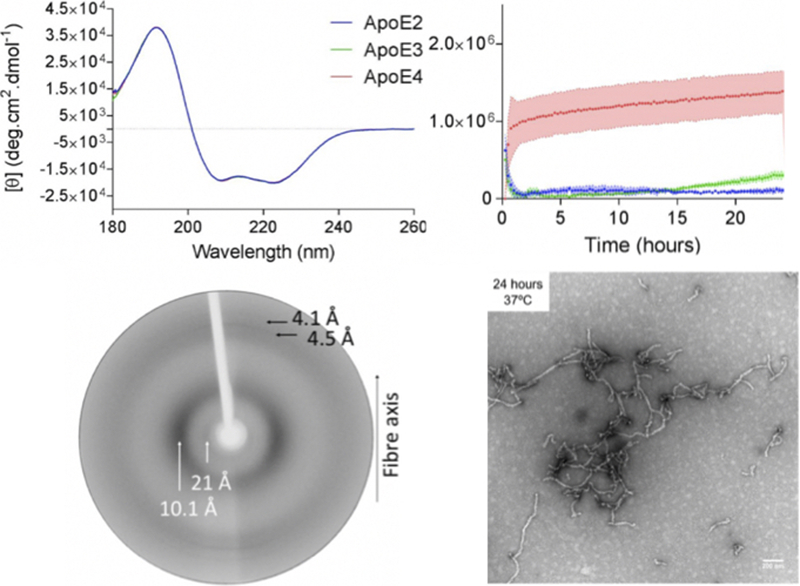

## Background

Alzheimer's disease (AD) is the most prevalent dementia, and the sporadic, late-onset form comprises 95% of all AD cases. Diagnosis is based on the observation of specific brain pathology of extracellular amyloid plaques composed of amyloid-beta (Aβ) peptide and intracellular neurofibrillary tangles formed by tau [1]. The biggest risk factor for developing AD is age, but the ε4 variant of the apolipoprotein E (*APOE*) gene is the strongest genetic risk factor for the development of late-onset AD. The human *APOE* gene encodes three major protein isoforms, ApoE2, ApoE3 or ApoE4, that differ from one another at amino acid positions 112 and 158. While E2 contains two cysteine residues, E3 contains a cysteine and an arginine, and E4 contains two arginine residues at both sites, respectively [2]. ApoE4 presents a risk in a gene dose-dependent manner, with ε3/ε4 heterozygotes having a risk increased by three times and ε4/ε4 homozygotes having up to 15 times more chance of developing AD [3]. ApoE3 is the most common isoform, associated with a neutral risk. ApoE2 is under-represented in the population but is thought to be associated with a lower propensity for AD [4].

ApoE is a predominantly α-helical protein with 299 amino acids that is mainly produced in the liver and by astrocytes in the brain, as well as neurons particularly under stress conditions [5], [6]. ApoE is a component of lipoprotein particles [7] and associates with cholesterol, triglycerides and phospholipids [8]. It is found in the central nervous system participating in high-density lipoproteins and in plasma associated with very low-density lipoproteins, chylomicron remnants and high-density lipoprotein [9]. It has been suggested that the ApoE isoforms have different lipid binding preferences and that lipid binding may be mediated by a conformational change of the monomer [10]. The ApoE isoform amino acid substitutions are presumed to affect ApoE structure and function and have been suggested to be responsible for ApoE4 homozygotes increased risk for AD [11]. However, the mechanism(s) whereby ApoE4 confers an increased risk of developing AD, whether by a loss-of-function or a gain-of-toxic-function, remains poorly understood. It has been shown that ApoE forms complexes with Aβ through regions within their heparin-binding sites *in vitro* [12] and was found to be associated with Aβ senile plaques in human brain [13] as well as with neurofibrillary tangles [14]. Histopathological examination of post-mortem AD brains found a positive correlation between plaque density and dosage of the ε4 allele [14], [15], [16], and positron emission tomography studies matched these findings with ε4 carriers having higher Aβ deposition compared to non-carriers [17], [18]. ApoE4 has therefore been suggested to enhance Aβ fibrillization and reduce Aβ clearance, which is thought to contribute to AD progression [19]. Especially, aggregated forms of ApoE4 seem to enhance Aβ fibrillization and antibodies that specifically recognize non-lipidated and aggregated ApoE have been shown to reduce Aβ deposition in APPPS1-21/APOE4 mice [20]. Recent work has also shown that human ApoE4 transgenic animals have increased deposition of tau compared to ApoE3 human transgenics [21] indicating a link to accumulation of neurofibrillary tangles. In addition, human-induced pluripotent stem cell-derived neurons expressing ApoE4 were shown to have higher levels of tau phosphorylation as well as increased degeneration of GABAergic neurons that could be rescued by gene editing and converting ApoE4 into ApoE3 [22].

The NMR structure of full-length ApoE3 reveals a large, predominantly α-helical globular protein, with a flexible C-terminal region composed of a short helix (270–277) followed by an unstructured tail (residues 277–299) [23]. The structures for N-terminal domains for ApoE2 and ApoE3 have been solved by x-ray crystallography [24], [25] and show α-helical bundles, which map closely to the N-terminal domain of E3. It has been proposed that the amino acid substitutions lead to lowered thermal and chemical stability of ApoE4 and higher propensity to form stable intermediate unfolding states characteristic of molten globules arising from the formation of a salt-bridge that links the C and N-terminal domain in ApoE4 [26]. A potential consequence of this instability would be an enhanced proteolysis of ApoE4 in neurons, generating fragments that may interfere with cytoskeletal components such as tau protein and neurofilaments [27, 28]. Decreased stability and molten globule formation has additionally been suggested to drive ApoE4 to form toxic, fibril-like oligomers from full-length protein, whereas ApoE2 and ApoE3 do not appear to form high-molecular-weight species [29].

Here we conducted an in-depth comparison of the structure and stability of the three recombinantly expressed and purified ApoE isoforms using state-of-the-art biophysical techniques and explored ApoE4's intrinsic property to oligomerize and aggregate. In contrast to previous studies [25], [26], we show that the three recombinant isoforms share very similar quaternary, tertiary and secondary structures and thermal and chemical stability. We reveal that recombinant ApoE4 forms fibrillar structures, however, the resulting curvilinear fibrils are fragile and do not share the typical characteristics of cross-β amyloid. Furthermore, to investigate the ApoE4 dose-effect, we explored the effect of co-assembly of ApoE3 and E4. The presence of ApoE3 resulted in decreased rate of ApoE4 self-assembly and an inhibition of elongation to long filamentous structures.

## Results

### All three ApoE isoforms show similar size and shape

**Fig. 1:**
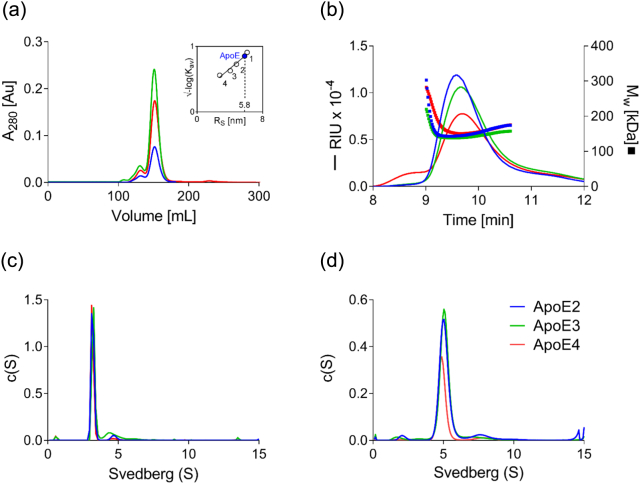
Characterization of recombinant ApoE2, E3 and E4. (a) The SEC elution pattern of ApoE isoforms on the Superdex 200 column as a function of absorbance at 280 nm *versus* the elution volume. All ApoE isoforms have a minor elution peak at 131 mL and a major at 151 mL suggesting the presence of different oligomeric species. A hydrodynamic radius of 5.8 nm, as well as a diffusion coefficient of 2.47 × 10^−7^ cm s^−1^ is calculated by calibration of the Superdex 200 column (inlet) using commercially available protein standards (1, ferritin; 2, aldolase; 3, conalbumin; 4, ovalbumin). A frictional ratio *f*/*f*_0_ = *R*_S_/*R*_min_ of 1.71 is calculated, which suggest moderate elongation of ApoE [31]. (b) SEC-MALS plotted with the differential refractive index (RIU; line) are shown as a function of elution time for ApoE2, ApoE3 and ApoE4. Inset shows the calculated molecular weight (MW) across the peak. All ApoE isoforms have identical elution volumes, and a MW of 145, 139 and 151 kDa is calculated at elution peak for ApoE2, ApoE3 and ApoE4, respectively. (c and d) AUC showing continuous *c*(*S*) size distributions in size exclusion buffer [20 mM Hepes, 300 mM NaCl and 10 % (v/v) glycerol pH 8.0; (c)] and in 20 mM PB (pH 7.4; (d). No difference between ApoE isoforms is observed in either buffer condition. A major species with a sedimentation coefficient *S* at 3 and 5 is observed in size exclusion and PB buffer, respectively. The difference in sedimentation coefficient between buffers is due to the presence of glycerol in the SEC buffer.

We have developed a method to produce and maintain full-length ApoE proteins recombinantly (see Materials and Methods and [30]), and it was therefore necessary to first fully characterise these proteins. Initial purification confirmed that ApoE2, E3 and E4 have identical mobilities of 34 kDa by non-reducing sodium dodecylsulfate (SDS)–polyacrylamide gel electrophoresis (PAGE; Fig. S1). Gel filtration and size exclusion chromatography (SEC) coupled with multi-angle light scattering (SEC-MALS) showed multimerization of all three proteins consistent with a tetramer in solution with a molecular weight (MW) of 139–151 kDa (Fig. 1a and b, Table 1). A hydrodynamic radius of 5.8–6.5 nm was calculated for each isoform, and a frictional ratio *f*/*f*_0_ above 1.7 suggests elongated shape for the homotetramers (Table 1). Analytical ultracentrifugation (AUC) was used to investigate the size and shape in solution in further detail. As we extensively dialyzed or buffer exchanged ApoE isoforms into phosphate buffer (PB) prior to stability and aggregation studies, we were interested if dialysis affected ApoE oligomerization in solution. We therefore compared sedimentation in the original size exclusion buffer and in PB after dialysis. AUC revealed no differences in sedimentation velocity between the three isoforms in either buffer (Fig. S3, Fig. 1c and d, Table 2a), and the major species was characterized with a molecular mass of 130 kDa (Table 2a).

**Table 1:** Parameters evaluated by gel filtration and SEC-MALS

		ApoE2, ApoE3 and ApoE4
Gel filtration^a^	Mw (kDa)	*333*
	*R*_s_ (nm)	5.8
	*f*/*f*_0_ = *R*_S_/*R*_min_	1.71
	*D* (cm^2^ s^−1^) × 10^−7^	2.47


		ApoE2	ApoE3	ApoE4
SEC-MALS^a^	Mw (kDa)	144	139	151
	*R*_s_ (nm)	6.55	6.4	6.81
	*f*/*f*_0_ = *R*_S_/*R*_min_	1.93	1.88	2.00
	*D* (cm^2^ s^−1^) × 10^−7^	3.78	3.88	3.64

a Values calculated at peak elution volume.

**Table 2a:** Sedimentation analysis of recombinant ApoE isoforms

Buffer		Frictional ratio^a^	Sedimentation velocity (*S*)^b^	Estimated MW (kDa)^b^	Peak integration (%)^b^	Overall RMSD value^c^
SEC	ApoE2	1.75	3.11	122	~ 90.7	0.003370
	ApoE3	1.77	3.26	129	~ 70.3	0.003085
	ApoE4	1.8	3.11	127	~ 89.5	0.002772
PB	ApoE2	1.77	5.07	127	~ 84	0.003275
	ApoE3	1.81	5.10	128	~ 88	0.002749
	ApoE4	1.74	5.23	130	~ 86.5	0.002789

a Best-fitt frictional ratio used to calculate continuous size distribution.

b Corresponding to the main sedimentation peak.

c The RMSD provide evidence for the goodness of the fit. Sedimentation analysis of recombinant ApoE isoforms shows that all three ApoE isoforms exist in a tetrameric form in size exclusion buffer [SEC; 20 mM Hepes, 300 mM NaCl, 10 % (v/v) glycerol; pH 8.0] and in 20 mM PB (pH 7.4) according to their estimated MW in solution.

**Fig. 2:**
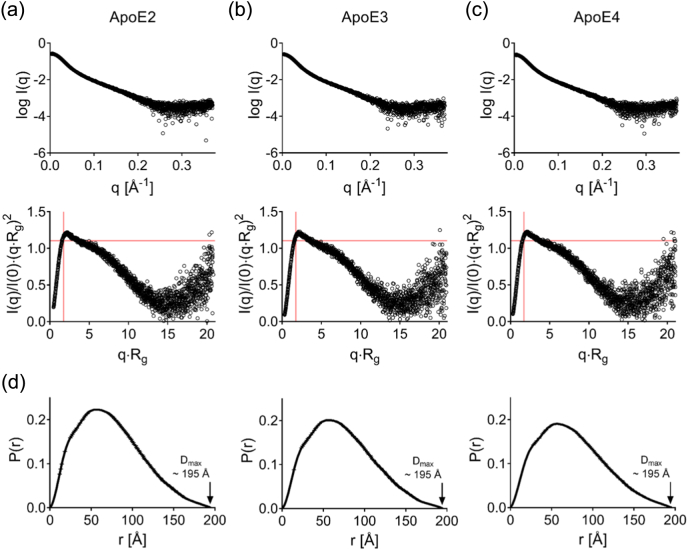
SAXS. X-ray scattering curves and the dimensionless Kratky plot (ScÅtter) for ApoE2 (a), ApoE3 (b) and ApoE4 (c) are shown, as well as their corresponding pair distance distribution function (d) *P*(*r*). (a–c) All ApoE isoforms have identical scattering profiles and adopt an extended conformation in solution with some intrinsic level of flexibility as assessed by the dimensionless Kratky plot. (d) This extended conformation is seen in the *P*(*r*) distribution, respectively, and a maximal dimension *D*_max_ of approximately 19.5 nm (195 Å) is determined for each isoform.

Small-angle x-ray scattering (SAXS) was used to explore whether the tetramers of the ApoE isoforms differ in dimensions and shape. All three proteins exhibited identical scattering profiles indicating that they are very similar in shape (Fig. 2a–c). A radius of gyration (*R*_G_) of 5.6 nm (56 Å) was calculated using the Guinier approximation and comes close to the hydrodynamic radius determined in the other techniques (Table 2b). The Kratky plot indicates that ApoE consists of several domains that are tethered by linkers with extended conformation. This elongated shape is reproduced in the pair distance distribution function and fits to a maximal dimension of approximately 19.5 nm (195 Å) (Fig. 2d).

**Table 2b:** Evaluation of the SAXS data by ATSAS and ScÅtter

	ApoE2		ApoE3		ApoE4	
	ATSAS	ScÅtter	ATSAS	vScÅtter	ATSAS	ScÅtter
Gunier analysis						
Guinier *R*_g_, (Å)	56.35 ± 0.15	56.34 ± 0.63	56.69 ± 0.14	56.81 ± 0.59	57.64 ± 0.15	57.69 ± 0.66
Gunier *I*(0), arbitrary units	0.25 ± 0.00049	0.28 ± 0.00044	0.25 ± 0.0004	0.25 ± 0.00034	0.24 ± 0.00041	0.24 ± 0.0063
*P*(*r*) analysis (manual)						
*P*(*r*) *R*_g_, (Å)	58.20 ± 0.08	57.56 ± 1.88	58.56 ± 0.088	57.78 ± 1.88	59.33 ± 0.067	58.46 ± 1.33
*P*(*r*) *I*(0), arbitrary units	0.28 ± 0.00035	0.27 ± 0.0094	0.25 ± 0.32	0.25 ± 0.0086	0.24 ± 0.00028	0.24 ± 0.0063
*D*_max_, (Å)	194.5	194.5	195	195	195.5	195.5
*q* range, (Å^−1^)	0.0089 - 0.2513	0.0083 - 0.2461	0.0056 -0.2414	0.0054 - 0.2394	0.0058 - 0.235	0.0056 - 0.2351
GNOM total estimate	0.92	–	0.922	–	0.929	–
χ^2^ (S_k2_)	–	1.17 (0.33)	–	0.88 (0.37)	–	1.02 (0.42)
*P*(*r*) analysis (Autognom)						
*P*(*r*) *R*_g_, (Å)	58.48 ± 0.12	–	58.69 ± 0.11	–	59.52 ± 0.13	–
*P*(*r*) *I*(0), arbitrary units	0.28 ± 0.00046	–	0.25 ± 0.00037	–	0.2415 ± 0.00039	–
*D*_max_, (Å)	204.68	–	203.65	–	207.92	–
*q* range, (Å^−1^)	0.0054 – 0.1419	–	0.0054 – 0.1401	–	0.0056 – 0.1383	–
GNOM total estimate	0.896	–	0.899	–	0.856	–
Volume of correlation (*V*_c_) and *M*_W_ estimate						
Volume of correlation *V*_c_, (Å^2^)^a^	–	1062	–	1068	–	1103
*M*_W_ estimate (kDa)^b^	–	159 ± 5	–	160 ± 5	–	169 ± 4

Parameters were calculated for comparison in ATSAS 2.8.2 and ScÅtter 3.1R.

a *P*(*r*)-based *V*_c_.

b Mass estimates based on *P*(*r*) *R*_g_ and *V*_c_[2].

Together AUC, SEC-MALS and SAXS show that the three ApoE isoforms are similar in size, shape and multimerization, and all form tetrameric species in solution.

### ApoE2, E3 and E4 are conformationally similar and show only marginal differences in stability

**Fig. 3:**
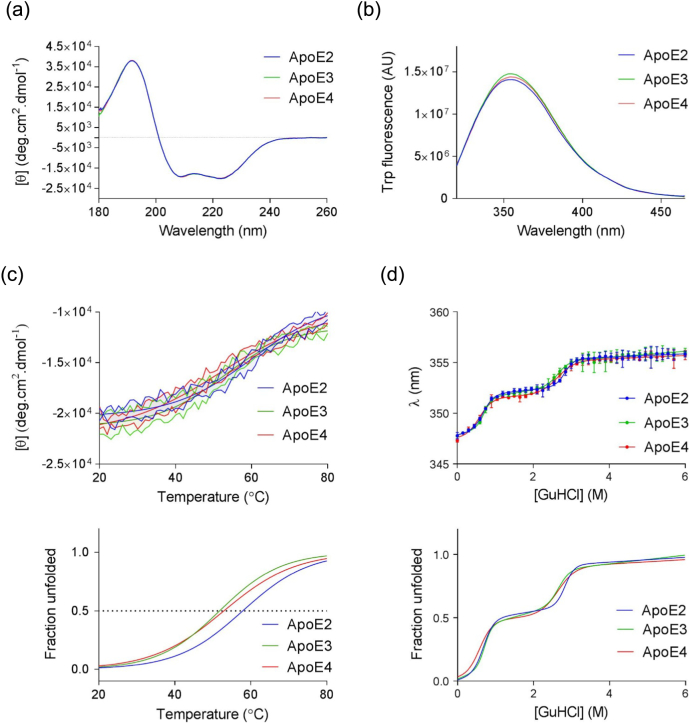
Conformation and stability of ApoE isoforms. (a) Far UV CD spectra of ApoE isoforms (25 μM in 20 mM PB, pH 7.4, 21 °C) showing comparable α-helical content. Secondary structure analysis was conducted with CONTIN/LL [32], [33] at DichroWeb [34], [35], [36] using the reference data set 6 [37], and results can be found in Table 3. (b) Intrinsic tryptophan fluorescence (excitation at 295 nm) indicates that all three isoforms have a similar tertiary structure in PB. (c) Changes in helical content were followed at 222 nm with increasing temperature. A Boltzmann sigmoidal equation was fitted to the data, showing the order of stability E2 >> E3 >= E4 (i). The fraction unfolded of each protein calculated from the fitted curves was also plotted against temperature (ii). Tm (°C) for each isoform, the temperature at which there was 50% change in α-helical content, estimated from the fit denaturation curves can be found in Table 4a. Comparable α-helical content at 37°C were also reported in Table 4a, and fraction unfolded at 37° C is shown in Table 4b. (d) GuHCl chemical denaturation of recombinant ApoE isoforms plotted showing the wavelength of maximum fluorescence *λ*_max_ at an excitation of 292 nm. Experimental data points (closed circles) are shown. (i) A three-state unfolding model was fitted to the data; apparent midpoint GuHCl concentrations can be found in Table 4b. (ii) Calculated fraction unfolded for each isoform from the fitted three-state unfolding model is shown. ApoE2 (blue connective line), ApoE3 (green connective line), ApoE4 (red connective line).

Circular dichroism (CD) spectroscopy and tryptophan fluorescence were used to probe potential differences in secondary and tertiary structures of the three isoforms. Negligible spectral differences were observed, suggesting no major conformational differences between the three proteins (Fig. 3a and b). Analysis of CD spectra using Dichroweb [34, 35, 36] showed that the three isoforms all possessed around 58% α-helical content (Table 3), with no significant differences between them (one-way ANOVA: *F*(2,9)=4.197, *p* > 0.05).

**Table 3:** Secondary structure analysis of recombinant ApoE isoforms from CD spectroscopy data^a^

	α-Helix (%)	β-Strand (%)	β-Turn (%)	Random coil (%)	NRMSD (%)
ApoE2	57.3	3.5	14.1	25.3	0.0178
ApoE3	59.5	3.3	13.0	24.2	0.0190
ApoE4	58.4	3.4	13.5	24.7	0.0184

The normalized root mean square deviation (NRMSD) is an estimate of the goodness-of-fit (NRMSD < 0.1 is a necessary but not sufficient condition.

α-helical content was similar between the three ApoE isoforms (within experimental error).

a Calculated by CONTIN program at DichroWeb (http://dichroweb.cryst.bbk.ac.uk/html/home.shtml).

Folding stability of ApoE proteins was examined using thermal and chemical denaturation and monitored using CD and tryptophan fluorescence. Thermal denaturation monitored using CD at 222 nm showed a sigmoidal unfolding curve for all three isoforms. A phenomenological Boltzman sigmoidal curve was fitted to the data and revealed that ApoE3 and ApoE4 have similar melting temperatures of 52.39 °C and 51.32 °C, respectively, compared to the apparent higher melting temperature for ApoE2 of 60.28 °C (Fig. 3c(i)). However, despite these slight differences in the apparent mid-point melting temperature, no differences were observed at physiological temperature of 37°C. One-way ANOVA provided evidence of no significant difference in the mean ellipticity at 222 nm between the three isoforms at 37 °C (*F*(2,9) = 0.9426, *p* = 0.4249; Table 4a). The curves showing fraction unfolded protein for each isoform calculated based on the fitted sigmoidal curves allow direct comparison of the estimated temperature unfolding transitions (Figs. 3c(ii) and S4A), showing similar thermal denaturation profile for all three isoforms. Furthermore, the fraction unfolded at 37°C showed that there were no significant differences between the three isoforms at physiological temperature, consistent with the mean ellipticity at 222 nm at the same temperature (Table 4b).

**Table 4a:** Thermal denaturation parameters from Boltzmann sigmoidal equation curve

	Tm (°C)	[Θ]_222 nm_ at 37 °C (deg cm^2^ dmol^−1^)
ApoE2	60.28 ± 1.18	− 19181 ± 649
ApoE3	52.39 ± 1.92	− 19809 ± 961
ApoE4	51.32 ± 4.32	− 19092 ± 773

Tm (°C) represents the temperature at which there was 50% change in α-helical content.

Non-linear best-fit parameters were determined on GraphPad Prism.

**Table 4b:** Fraction unfolded of ApoE at 37 °C

	Fraction unfolded at 37 °C	%Fraction unfolded
ApoE2	0.1 ± 0.07	10
ApoE3	0.13 ± 0.03	13
ApoE4	0.17 ± 0.06	17

Fraction unfolded of ApoE at 37°C was calculated from the fraction unfolded curves given by Eq. (7) (Fig. 3c). Non-linear best-fit parameters were determined using GraphPad Prism.

Chemical denaturation in guanidium hydrochloride (GuHCl) was performed in reducing conditions (1 mM dithiothreitol), and the wavelength of maximum fluorescence emission intensity *λ*_max_ was plotted against corresponding concentrations of GuHCl. All three denaturation curves demonstrate multi-phasic behavior and a two-state unfolding model did not fit the denaturation data. Instead, a three-state unfolding model was fitted to the data (Fig. 3d(i)), suggesting the existence of at least three different states of the protein, and the existence of one or more intermediate states. The data are, therefore, consistent with the separate unfolding of two protein domains. From the fitted curves, GuHCl concentrations corresponding to the midpoint of each transition were calculated (Table 4c). Similar to the thermal denaturation study, fraction unfolded curves were calculated for each isoform in order to directly compare the shape of the chemical denaturation profile (Fig. 3d(ii)). The transition from a folded state to an intermediate state occurs at a slightly lower GuHCl concentration for ApoE4, suggesting that the first domain to unfold denatures marginally more easily for ApoE4 than ApoE2 and ApoE3 (0.58 M *versus* ~ 0.71 M). ApoE3 and ApoE4 show similar unfolding of the second domain, while ApoE2 is more stable (~ 2.65 M *versus* 2.84 M). Overall, the observed increased chemical denaturant concentration and temperature needed for ApoE2 to unfold compared with ApoE3 and E4 infers that ApoE2 has a slight increased resistance to denaturation compared to E3 and E4, although the difference in apparent stability is marginal (Table 4c).

**Table 4c:** Chemical denaturation parameters from double sigmoidal curve

	[GuHCl]_50,I_ (M)	[GuHCl]_50,U_ (M)
ApoE2	0.69 ± 0.03	2.84 ± 0.04
ApoE3	0.71 ± 0.03	2.66 ± 0.07
ApoE4	0.58 ± 0.04	2.65 ± 0.08

[GuHCl]_50, I_ is the concentration needed for 50% of the protein to unfolded to an intermediate state. [GuHCl]_50, U_ is concentration needed for 50% of the intermediate state to reach the unfolded state. Non-linear best-fit parameters were determined using GraphPad Prism.

### ApoE4 assembles to form non-amyloid, native-like fibers

**Fig. 4:**
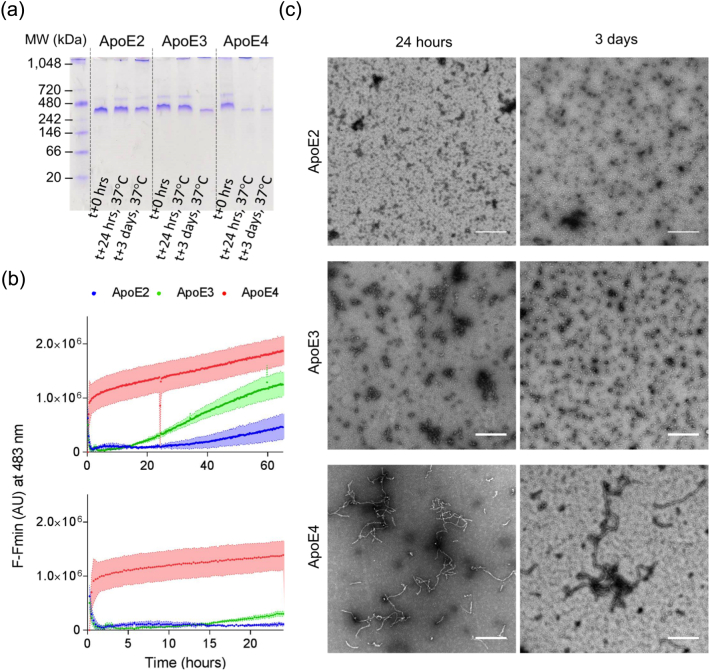
Self-assembly of ApoE4 but not ApoE2 or ApoE3. (a) Native gel shows the formation of higher oligomeric species for ApoE4 (> 1048 kDa) but not E2 and E3 after incubation at 37°C for 24 h. (b) Kinetics of ApoE self-assembly (ApoE2, blue; ApoE3, green; ApoE4, red) was monitored by recording changes in ThT fluorescence intensity at 483 nm over 3 days. A comparison between the three isoforms was established by looking at adjusted ThT fluorescence. Changes in ThT fluorescence varied with each isoform, with a faster increase observed for ApoE4. After 3 days, ApoE3 and ApoE4, but not ApoE2, show changes in ThT fluorescence (top panel). The bottom panel is a close-up on the first 24 h of assembly. The length of the lag phase was different for each isoform, with ApoE2 > ApoE3 >> ApoE4. Envelopes correspond to the standard error of the mean (SEM). (c) TEM of negatively stained ApoE isoforms after incubation for 24 h (left side) and 3 days (right side) at 37 °C shows no fibril formation of ApoE2 and ApoE3. ApoE4 self-assembles to form long, curved fibrils. ApoE3 formed round oligomeric species after a 3-day incubation. The scale bars represent 500 nm.

To further investigate the behavior of the ApoE isoforms, the three proteins were incubated in 20 mM PB at 37 °C for 24 h. Native PAGE showed that ApoE2 and E3 ran consistently at a similar mobility at 0 h and after a 24-h incubation. However, ApoE4 formed higher MW species prior to and following incubation. By 24 h, the majority of the ApoE4 protein formed higher oligomers that did not run through the gel indicating oligomerization of the ApoE4 protein (Fig. 4a). Thioflavin T (ThT) fluorescence assay is frequently used to monitor molecular self-assembly in solution, and although it is often utilized to monitor amyloid formation [38], it is not specific for β-sheet structure or for amyloid [39], [40]. ThT was excited at 440 nm, and its fluorescence intensity was followed at 483 nm. The change in fluorescence adjusted to baseline displayed a rapid increase in intensity at 483 nm for ApoE4 (Fig. 4b(i and ii)), and an extended lag phase for ApoE3 before any changes were detected (Fig. 4b(ii). There was very little change in fluorescence for ApoE2 up to 24 h and only minor increase up to 60 h (Fig. 4b). After a 3-day incubation at 37 °C, the ThT fluorescence increased further for ApoE4, while no further differences in ThT intensity were observed for ApoE2 (Fig. S5). ThT fluorescence data indicate fibrillization of ApoE4, while only minor changes and no changes were observed for ApoE3 and E2, respectively. TEM was used to observe morphological changes with time. TEM of ApoE2 showed some small amorphous and some round species after 24-h and 3-day incubation, respectively (Fig. 4c). Small round species were observed at 24 h for ApoE3 that increased in size over time and became slightly elongated after 3-day incubation (Fig. 4c). In contrast, ApoE4 showed fibrillar structures with a curvy appearance at 24 h (Fig. 4c).

**Fig. 5:**
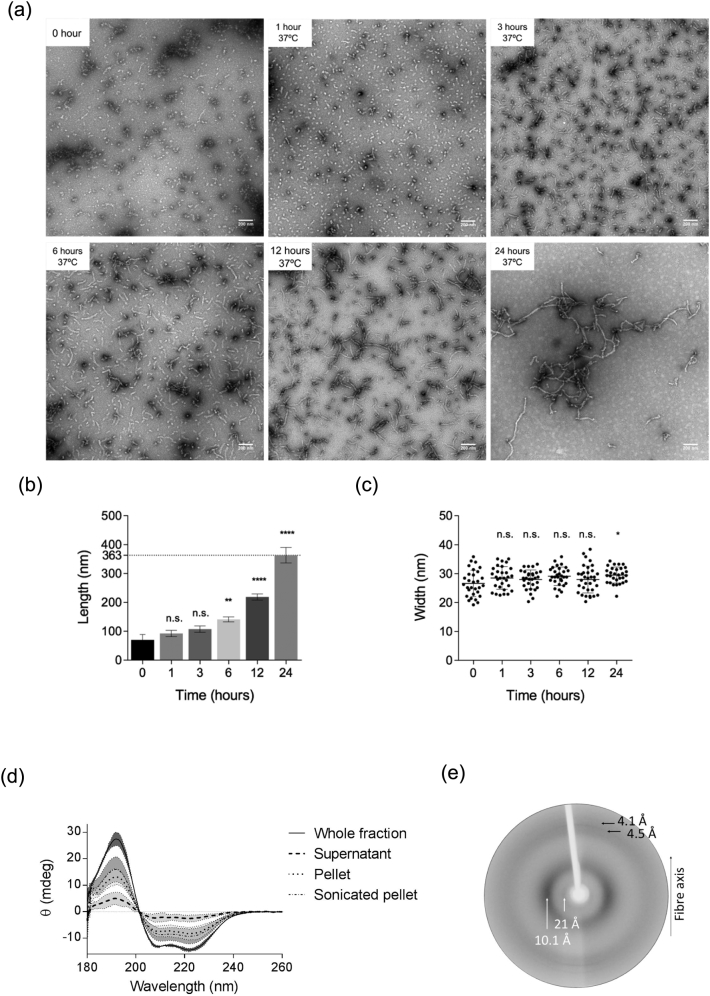
Characterization of ApoE4 fibrils. (a) Transmission electron micrographs of negatively stained ApoE4 (25 μM in 20 mM PB, pH 7.4) monitored over 24 h at 37 °C. The scale bar represents 200 nm. ApoE4 self-assembly was characterized by measuring changes in length (b) and width (c) of the fibrils. Changes in width were non-significant; however, with increasing incubation times, ApoE4 fibrils become significantly longer (average length of 363 nm; one-way ANOVA: ****p < 0.0001, *F* = 180.9). (d) CD spectra show retention of α-helical secondary structure after assembly (whole fraction). Fibrils in the pellet were separated from the supernatant to confirm that their secondary structure is not masked by protein in the supernatant. Fibrils in the pellet showed an α-helical conformation. (e) X-ray fiber diffraction pattern obtained from partially aligned ApoE4 fibrils after 24-h incubation at 37 °C showing positions of diffraction signals on the meridian (vertical) and equatorial (horizontal) axes.

To further explore the assembly of ApoE4, TEM was used to monitor the size and morphology of the assemblies at different time points up to 24 h. The electron micrographs show accumulation of small rounded species after only 1-h incubation. The formation of short, curved filaments is detected by 3-h incubation, which develop into curvy-linear filaments by 6 h and elongate into extended, curvy filaments by 24 h (Fig. 5a). Measurement of the average length of the filaments (Fig. 5b) confirms that the filaments extend in length, while the diameter of the structures remains invariant over 24 h, with an average length of 363 nm and width of 30 nm (Fig. 5b and c). Close inspection of the filamentous structures shows that they have a granular appearance and do not share the twisted and smooth appearance generally observed for amyloid fibrils [41], [42], [43]. Fibril formation appears to arise from the end-to-end fusion of the small spherical species, making this unit the apparent smallest building block of the structure.

CD and x-ray fiber diffraction experiments were conducted to investigate the nature of ApoE4 fibrils and to investigate whether these assemblies are amyloid-like in structure and undergo the expected conformational change to β-sheet [29]. CD spectra collected for ApoE4 fibrils incubated for 24 h showed a spectrum with minima at 208 and 222 nm consistent with a predominantly α-helical secondary structure content (Fig. 5d), of comparable shape and intensities to that of non-assembled ApoE4 (Fig. 3a). To ensure that any remaining soluble protein does not dominate the spectra, the sample was centrifuged at high speed to separate supernatant and pellet fractions. The CD data for the resolubilized pellet fraction show that E4 retains its α-helical structure after incubation at 37 °C for 24 h, and that there is almost no soluble protein left given the very small CD signal in the supernatant fraction (Fig. 5d). The fiber pellet was sonicated to ensure that protein was resuspended sufficiently and the spectrum continued to demonstrate α-helical content.

To examine the molecular structure of the mature ApoE4 filaments, 100 μM ApoE4 was incubated for 24 h and aligned to form a partially-aligned fiber bundle. The x-ray fiber diffraction pattern showed a sharp meridional diffraction signal at 4.5 Å with a weaker diffraction signal at 4.1 Å. On the equator, a diffuse, strong signal was observed at 10.1 Å and a weaker reflection at approximately 21 Å (Fig. 5e). Comparison of the relative intensities shows that the meridional reflections are weaker than those on the equator, which is dissimilar to those usually observed for amyloid fibril patterns where signals arise from cross-β structure at 4.7 and 10 Å on perpendicular axes [44]. The diffraction data obtained support the CD data described above, showing that the ApoE4 retains an α-helical conformation in the fibers, which is similar to the structure in the soluble protein.

### ApoE3 and ApoE4 influence the assembly of one another

**Fig. 6:**
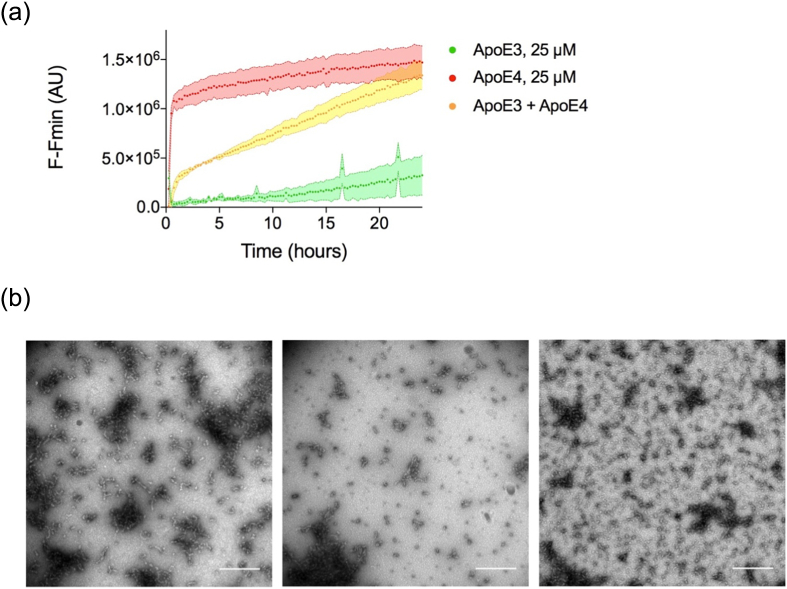
ApoE3 inhibits ApoE4 fibril formation. (a) Adjusted ThT fluorescence at 483 nm was monitored over the course of 24 h, 37 °C for ApoE3 alone (25 μM), ApoE4 alone (25 μM) and ApoE3 plus ApoE4 (both 12.5 μM for a total ApoE concentration of 25 μM) in PB (pH 7.4). (b) Transmission electron micrographs showed a heterogeneous population, with the presence of small round and small amorphous species, but no mature fibrils when E4 is incubated with E3 in a 1:1 ratio. The scale bar represents 500 nm.

ApoE4 alone is able to self-assemble to form non-amyloid-like filaments. However, the majority of individuals with an ε4 allele are heterozygous and have an ε3 allele. To investigate the influence of ApoE3 presence on ApoE4 assembly and vice versa, ApoE3 was incubated with ApoE4 over the course of 24 h to investigate whether they could enhance/seed or inhibit assembly. ApoE3 and ApoE4 were each at 12.5 μM to give a combined ApoE concentration of 25 μM. Assembly over time was compared to assembly of ApoE3 or ApoE4 alone at 12.5 μM (not shown) or 25 μM. Figure 6a shows that ApoE3 reduced the rate of assembly of ApoE4, measured by ThT fluorescence; the ThT kinetics of the mixed sample did not resemble that of E3 alone either. TEM micrographs were produced for the mixed sample and showed the presence of small round and small amorphous species instead of the long mature fibrils observed for ApoE4 alone (Fig. 6b).

## Discussion

The manner in which *APOE* genotype leads to an increased risk for late-onset AD remains unclear. Some studies have proposed that the three ApoE isoforms differ in their structure and consequently function, which may explain ApoE4's differential effects on AD pathogenesis [3], [45]. Modeling and fluorescence resonance energy transfer studies have been used to provide evidence of a more open conformation for ApoE4 compared to ApoE3 [11], [46], [47], suggesting that the N-terminal region of ApoE4 is less well tethered to the C-terminal domain than for ApoE3. Others have pointed to differences in stability and conformation [29], [48] and have suggested that ApoE4 is less stable and more prone to aggregation to form amyloid-like fibrils [29]. *In vivo*, ApoE is glycosylated and also lipidated [49]. While we have explored the structural conformations adopted by recombinant non-modified ApoE, previous studies have shown that the recombinant ApoE produced in *Escherichia coli* adopts a very similar conformation and folding to protein produced in adenovirus [50]. Further studies will be necessary to explore the behavior of lipidated proteins.

Here we have investigated the size, oligomerization state and shape of recombinant ApoE isoforms in aqueous solution. Our results are in agreement with previous observations [51] and confirm that all three isoforms form an elongated tetramer in solution. SAXS data provides additional information regarding the low-resolution structure of ApoE isoforms in solution and validates the elongated shape in solution with ApoE qualitatively resembling multiple domains that are tethered by flexible linkers. Identical x-ray scattering profiles between ApoE isoforms suggest no major structural differences in macromolecular architecture.

CD reveals that all three isoforms share a similar α-helical content at physiological pH, when measured at 21 °C. This is in contrast to other studies, which have reported differences in α-helical content between the isoforms at 15° C, with ApoE4 being the least α-helical protein [48]. Here, temperature and chemical denaturation studies revealed very small differences in stability towards unfolding for the three isoforms. Melting curves displayed a sigmoidal shape that is characteristic for two-state unfolding, with Tm(E2) ≥ Tm(E3) ≈ Tm(E4), which is in agreement with previously reported results in terms of order of stability [25], [48], [52]. However, it is also important to highlight that all three isoforms have the very similar secondary structure content and fraction unfolded at physiological temperature of 37 °C.

Chemical denaturation displayed multi-phasic unfolding curves for all three isoforms, consistent with the independent unfolding of the C-terminal and N-terminal domains as described by Morrow and colleagues [25]. While the curves cannot be used to fully deduce Δ*G*°_H2O_ and the slope *m*-value for each transition since the three-state model employed is empirical in this case, we interpret slight differences in the apparent denaturant concentration corresponding to the midpoint of each transitions. On the one hand, the transition from the folded to intermediate state was very similar for ApoE2 and ApoE3; however, it occurred at a lower denaturant concentration for ApoE4. On the other hand, transitioning from intermediate to unfolded was most similar between ApoE3 and ApoE4, whereas ApoE2 required a slightly higher denaturant concentration. Others have gone further with chemical denaturation studies by looking at the 10 kDa carboxy- and the 22 kDa amino-terminal domains, in conjunction to the corresponding full-length proteins. Data resulted in the attribution of the first transition to the unfolding of the 10-kDa carboxy fragment and the second to that of the 22-kDa amino-terminal domain, while reporting the same order of chemical stability [25], [48], [53].

The loss of stability of ApoE4 as measured by spectroscopic methods, albeit under extreme conditions such as high temperature or under denaturing conditions, may be related to its propensity to polymerize into filamentous structures. We showed here that ApoE4 forms filamentous structures after only 24-h incubation at physiological temperature, pH 7.4, while ApoE2 and ApoE3 remained soluble and globular under the same conditions. TEM reveals that the ApoE4 filaments have a polymeric appearance (beads on a string), and there is a clear hierarchical assembly of small spherical species to small, elongated fibrils and finally fibrils, which show identical diameters. CD shows that following incubation, the ApoE4 fibers retain their α-helical secondary structural content. The CD for the whole fraction is almost identical to ApoE4 prior to incubation. To probe whether the α-helical intensity arises from soluble, unassembled protein, we examined the CD from a sedimented sample. Both pellet and supernatant fractions show a spectrum consistent with high α-helical content. Furthermore, the intensity of the spectrum in the supernatant was very low, suggesting that the majority of the ApoE4 protein is found within the fiber containing pellet. This result contrasts with a small shift from α-helix to β-sheet previously described by Hatters *et* *al*. [29]. ThT fluorescence assay shows increasing intensity at 483 nm with time showing that the fibril formation course can be followed using this chemical rotor [39], [40]. Although ThT is well characterized as fluorescent probe for amyloid fibril formation, it is a molecular rotor that is not specifically sensitive to β-sheet structures. Amyloid binding studies have revealed that ThT may associate with aromatic groups including tyrosine ladders [54], [55]. Fluorescence intensity can increase in the presence of many polymeric molecules and in viscous solutions [54]. Furthermore, ThT is fluorescent in the presence of cross-α fibrous molecules revealing that its fluorescence does not rely on β-sheet structure [56].

X-ray fiber diffraction has been classed as a definitive diagnostic for amyloid fibrils, giving the classical amyloid cross-β diffraction pattern composed of a 4.76-Å meridional arising from hydrogen bonding β-strands running perpendicular to the fiber axis and a more diffuse equatorial signal that arises from β-sheet packing that accommodates side chains [57], [58], [59]. Here we revealed that the ApoE4 filaments do not give a cross-β pattern, instead diffraction shows a pattern that can be interpreted as α-helical rich polymer, consistent with the TEM and CD data. An α-helix has a repeat of 5.4 Å parallel to the axis of the helix (1.5 Å rise per residue, with 3.6 residues/turn) [60]. However, the structure of the ApoE molecule is not arranged with all helices arranged in a vertical array. 4.5 Å is a repeating unit along an extended polypeptide chain and was previously observed for dried peptide crystals [61], while 10 .1 Å approximates to the packing of helices in the vertically aligned ApoE structure. Furthermore, amyloid fibrils are characterized by their high stability and resistance to degradation. For example, amyloid formed by the Alzheimer's-related peptide is SDS resistant [62]. ApoE4 fibrils run as a monomer by SDS page (data not shown) and the ApoE4 species run on a native gel at similar mobility to ApoE3 and ApoE2. Many examples of native like polymers exist including pathologically related serpins such as α-1-antitrypsin [63] and functional polymeric proteins such as microtubules and actin.

The majority of individuals are heterozygous ε3/ε4, and this confers an increased risk for AD development, albeit lower than for ε4/ε4 individuals [64]. Here we investigated the aggregation potential for mixed population of ApoE3 and ApoE4 and revealed that E3 inhibits E4 fibril formation *in vitro,* leading to a mid-range aggregation kinetic for assembly*.* Therefore, the increased dose of ApoE4 in ε4/ε4 individuals leads to increased assembly compared to half dose of ApoE4 in those who posses ε3/ε4.

## Conclusions

The detailed studies conducted have revealed that recombinant ApoE2, ApoE3 and ApoE4 resemble one another at quaternary, tertiary and secondary structural levels. All three ApoE isoforms form elongated tetramers in solution, and each monomer is rich in α-helical conformation. There is no evidence that the aqueous proteins differ at a structural level. Furthermore, chemical and thermal denaturation studies reveal that the three proteins are similarly stable and follow comparable unfolding mechanisms by melting and by chemical denaturation. However, we have shown that ApoE4 has a higher propensity for polymerization at physiological temperature and pH, to form elongated fibrous structures which retain the native α-helical conformation. The propensity of ApoE4 to self-assemble may play an important role in the mechanism by which it increases susceptibility to develop AD, which impacts on ε4/ε4 individuals more severely than those with ε3/ε4.

## Materials and Methods

All materials were purchased from Sigma-Aldrich or Fisher Scientific at the highest purity available.

### Protein production

ApoE2, ApoE3 and ApoE4 were cloned, expressed and purified using methods detailed in the supplementary information. Briefly, a codon-optimized ApoE4 gene was cloned into a pET17b vector with a six-histidine tag, thioredoxin and HRV 3C protease cleavage site upstream. ApoE2 and ApoE3 genes were generated by site-directed mutagenesis using QuikChange (Agilent). Recombinant ApoE proteins were expressed in *E. coli* Rosetta2(DE3) cells and affinity purified using Talon® beads followed by HiTrap heparin affinity column and cleaved on column with PreScission protease. The ApoE proteins were eluted using a salt gradient and further purified using a HiLoad Superdex 26/600 200 pg size exclusion column. ApoE containing fractions were concentrated and stored at -80 °C.

### Gel filtration studies

The high-molecular-weight protein standard (GE Healthcare, no. 28-4038-42) was used to calibrate the HiLoad Superdex 26/600 200 pg column (GE Healthcare). All protein standards and dextran blue were dissolved in SEC buffer [20 mM Hepes, 300 mM NaCl, 10% (v/v) glycerol (pH 8.0)] according to the manufacturer's recommended concentrations (0.4 mg/mL ferritin, 4 mg/mL aldolase, 3 mg/mL conalbumin, 3 mg/mL ovalbumin), and 1.6 mL of the protein calibration mixture was applied onto the column at a flow rate of 1.5 mL/min. Stokes radius (size) values were calculated on the basis of ApoE elution volume (Eq. (1))

(1) $$K\mathrm{av}=\frac{Ve-V0}{Vt-V0}$$

where *V*_e_ = elution volume for the protein, *V*_0_ = Superdex 200 void volume (112.26 mL), and *V*_t_ = total Superdex 200 bed volume (320 mL). *K*_av_ values were calculated for each protein standard and a standard curve generated by plotting the √(− log(K_av_)) as *y* values *versus* the Stoke radius of each standard as *x* values (ferritin 6.1 nm, aldolase 4.8 nm, conalbumin 4.04 nm, ovalbumin 2.75 nm). ApoE Stoke radius *R*_S_ was estimated with the following best fit linear Eq. (2):

(2) $$y=0.1058\;x+0.2397\;\left(R^{2}=0.9552\right)$$

ApoE diffusion coefficient *D* was estimated from its Stoke radius *R*_S_ by the following Eq. (3):

(3) $$D=\frac{\mathrm{kT}}{6\mathrm{\pi \eta R}s}$$

where *k* is the Boltzmann constant (1.38 × 10^−23^ m^2^ kg s^−2^ K^−1^), *T* is the experimental temperature (277.15 K) and *η* is the solvent viscosity (1.4181 × 10^−3^ Pa s) at temperature *T*.

The frictional ratio *f*/*f*_0_ = *R*_S_/*R*_min_ was calculated by assuming the minimal radius (*R*_min_) of a sphere that could contain the given mass of tetrameric ApoE (136,800 Da), where *R*_min_ [31] is defined as

(4) $$R_{\min}=0.066\;M^{\frac{1}{3}}\;\left(\mathrm{for}\;M\;\text{in Dalton},R_{\min}\;\text{in}\;\mathrm{nm}\right)$$

### Dialysis and buffer exchange

ApoE was either extensively dialysed overnight in 20 mM PB (16 mM Na_2_HPO_4_, 4 mM NaH_2_PO_4_) at pH 7.4 using Slide-A-Lyzer™ Dialysis Cassettes with a molecular weight cutoff (MWCO) of at 3.5 kDa (Thermo Fisher) or buffer exchanged using disposable Vivaspin® 500 centrifugal concentrators with an MWCO of 3 kDa or Vivaspin® 20 centrifugal concentrators with an MWCO of 3 or 10 kDa (Sartorius).

### Gel electrophoresis

Protein samples for denaturing one-dimensional SDS–gel electrophoresis were prepared in Laemmli sample buffer (Bio-Rad) to a final concentration of 3 μM, omitting the addition of any reducing agent. Samples were loaded into a 4%–20% TGX gel (Bio-Rad), and the gel was run at 120 V (constant voltage) for 80 min. Resolved proteins were stained using a Coomassie dye R-250 containing solution (Imperial protein stain, no. 24615; Thermo Scientific).

Protein samples for native PAGE were diluted to 3 μM in native sample buffer (Bio-Rad) and were directly loaded into a 4%–20% TGX gel (Bio-Rad). Gels were run at 120V for 120 min. Resolved proteins were stained using a Coomassie dye R-250 containing solution.

### SAXS and SEC-MALS

SAXS and MALS experiments were performed at the B21 beamline (Diamond Light Source, UK).

X-ray scattering was acquired with an x-ray wavelength of 1 Å on a Pilatus 2M detector at a distance of 3.9 m and a camera length of 4.036 m. ApoE isoforms at 10 mg/mL were delivered at 20 °C and a flow rate of 0.16 mL/min via an in-line Agilent high-performance liquid chromatography with a Shodex Kw-403 column and 20 mM Hepes and 300 mM NaCl (pH 8.0) as running buffer. In total, 620 frames were recorded and each frame exposed for 3 s. Buffer subtraction and averaging was performed in ScAtter version 3.1R. Data was analyzed in ScAtter version 3.1R and ATSAS 2.8.2 for comparison.

ApoE isoforms were diluted to 5 mg/mL in 20 mM Hepes, 300 mM NaCl and 1 mM TCEP (pH 8.0) for MALS experiments and delivered at RT via an in-line Agilent high-performance liquid chromatography with a Shodex Kw-403 column. Refractive increments (dn/dc) were determined by a Wyatt optilab T-rEX and scattering measured by a Wyatt Dawn Heleo with QELS. Data were analyzed using ASTRA version 6.1.7 (Wyatt).

### AUC

Sedimentation velocity experiments were carried out in a Beckman model XL-A analytical ultracentrifuge (Beckman Coulter, Fullerton, CA) at 20 °C using an AnTi60 rotor. ApoE isoforms (400 μL) were used at a concentration of 8 μM in 20 mM Hepes, 300 mM NaCl and 10% (v/v) glycerol (pH 8.0) or at a concentration of 8 μM in 20 mM PB (pH 7.4). Experiments were performed at a rotor speed of 40,000 rpm (128,794*g*), and absorbance at 280 nm was recorded every 25 min, at radial intervals of 0.003 cm. A continuous size c(S) distribution c(S) from the Lamm equation model in the range of 0.1S–15S was fit to the data using the program SEDFIT version 15.01b [65]. The confidence interval (*F*-ratio) was set to 0.95. Buffer viscosity and density were calculated in Sednterp version 20120828 BETA [66].

### CD spectroscopy

ApoE isoforms were diluted to a final concentration of ~ 25 μM in 20 mM PB (pH 7.4). The exact concentrations were confirmed in triplicate using a NanoDrop ND2000c spectrophotomer (Thermo Fisher) by measuring the absorbance at 280 nm and applying Beer-Lambert's law using an extinction coefficient of 44,460 L mol^−1^ cm^−1^ The averaged concentrations were recorded and used in calculations when needed.

FarUV CD data were collected using a Jasco-715 CD-spectrometer (Jasco, Goh-Umstadt, Germany) at different time points. Temperature was maintained at 21 °C using a Peltier controlled cell holder. All spectra were collected in 0.01-cm transparent quartz cuvettes (Starna Scientific, Essex, UK) in the range of 280–180 nm with a resolution of 0.1 nm and a bandwidth of 1 nm. Scanning speed was set to 50 nm/min, response time to 4 s and sensitivity to standard. Three spectra per sample were acquired and averaged to give a spectrum after subtracting blank spectra of the buffer. For each isoform, spectra were collected on protein from at least three different production batches and an average trace of [Θ] against wavelength was obtained using GraphPad Prism. The mean residue ellipticity ([Θ] in deg cm^2^ dmol^−1^) was calculated from the measured ellipticity Θ in mdeg at wavelength *λ* using Eq. (5):

(5) $$\left[\Theta \right]=\frac{\Theta }{10.n.C.l}$$

with *n* the number of amino acid bonds in the protein, *C* the concentration of the sample in mol L^−1^ and a path length of 1 cm.

Secondary structure of ApoE isoforms was calculated by deconvolution of CD spectra performed with CONTIN/LL [32], [33] at DichroWeb [35] using the reference spectra set 6 [37]. Results represent a mean of values from a minimum of three spectra per isoform. One-way ANOVA was performed to compare the α-helical content between the isoforms, using GraphPad Prism.

### Thermal denaturation using CD

ApoE isoforms were diluted to a final concentration of ~ 25 μM in 20 mM PB (pH 7.4). CD data were collected using a Jasco-715 CD-spectrometer (Jasco, Goh-Umstadt, Germany). Temperature was increased at a rate of 1°C/min from 20 °C to 80 °C using a Peltier-controlled cell holder. Thermal scans were acquired at a wavelength *λ* of 222 nm every 1.0 °C with a sensitivity of 100 mdeg, a response time of 1 s and a bandwidth of 1 nm. Thermal scans were acquired for proteins purified from at least three different production batches, and an average trace of [Θ] against temperature was obtained using GraphPad Prism. The unfolding was irreversible. A phenomenological Boltzmann sigmoidal curve with the following Eq. (6) was fitted to the averaged thermal denaturation curves:

(6) $$Y=\frac{\mathrm{Top}-\text{Bottom}}{1+e^{\frac{Tm-X}{\text{Slope}}}}$$

with Top = maximum Θ value, Bottom = minimum Θ value, Tm the temperature corresponding to 50% change in α-helical content and Slope = the steepness of the curve. The calculated thermal denaturation curve based on the fitted curves was also transformed into the fraction of the protein unfolded for comparison of protein transformation with the following Eq. (7):

(7) $$\text{Fraction unfolded}=\frac{{\left[\Theta \right]}_{N}-\left[\Theta \right]}{{\left[\Theta \right]}_{N}-{\left[\Theta \right]}_{D}}$$

with [Θ]_N_ the ellipticity of the protein at 20 °C corresponding to the folded state and [Θ]_D_ the ellipticity of the denatured protein at 80 °C.

### Intrinsic fluorescence measurements

Recombinant ApoE isoforms were diluted to 10 μM in 20 mM PB and incubated overnight at 4 °C. Fluorescence scans were acquired using SpectraMax i3 reader (Molecular dimensions). Excitation was set to 295 nm, and scans were collected with 1-nm increments between 320 and 465 nm. Excitation and emission bandwidths were set to 9 and 15 nm, respectively. The number of reading per well was set to 6. The photomultiplier tube voltage was set to high. Blank spectra of the buffer were subtracted to protein fluorescence scans. A minimum of three different production batches per isoform were used, and an average trace of fluorescence intensity against wavelength was obtained using GraphPad Prism.

### Chemical denaturation and data analysis

Recombinant ApoE isoforms at ~ 0.05 mg/mL in 20 mM PB and 1 mM dithiothreitol were incubated overnight at 4 °C with increasing concentrations of GuHCl (0–6 M) [25]. Measurements were obtained with a Varian Cary Eclipse spectrophotometer (Varian Ltd., Oxford, UK) and quartz cuvette (1-cm path length; Starna, Essex, UK). Temperature was maintained at 20 °C using a Varian Cary temperature controller.

Tryptophan residues were selectively excited at 292 nm and emission was monitored between 310 and 400 nm; excitation and emission slits were set to 10 nm. Five acquisitions per concentration point were acquired at a scan speed of 90 nm/min using response time of 0.05 s. A minimum of three different production batches per isoform were used [67].

Wavelength of maximum emission (*λ*_max_) per GuHCl point was determined by peak fitting of the averaged emission spectra [48]. Denaturation curves were obtained by plotting the wavelength corresponding to the maximum fluorescence intensity against GuHCl concentration ([GuHCl]) on GraphPad Prism. The curves show two major transitions and can be described by a three-state unfolding model [68], as described in Eqs. (8), (9)

(8) Folded⇆Intermediate⇆Unfolded

(9) $$Y=\frac{Y_{F}\left(\left[\text{GuHCl}\right]\right)+Y_{U}\;\left(\left[\text{GuHCl}\right]\right)\cdot K_{\mathrm{app}}\;}{1+K_{\mathrm{app}}}$$

Equation (9) [68] was fitted to the data with *Y* corresponding to *λ*_max_, and *Y*_F_ and *Y*_U_ the signal corresponding to the folded (F) and unfolded (U) protein, respectively. *Y*_F_ and *Y*_U_ are assumed to be linearly dependent on the concentration of denaturant, as displayed in Eq. (10):

(10) $$Y_{X}=Y_{X}^{0}+m_{x}\left[\text{GuHCl}\right]$$

with *X* corresponding to either the F or U state and *Y*_*x*_^0^ the *λ*_max_ at either 0 M GuHCl or 6 M. In Eq. (9), *K*_app_ corresponds to the apparent guanidine-dependent equilibrium constant of the denaturation process, defined by Eq. (11):

(11) $$K_{\mathrm{app}}=\frac{K_{1}K_{2}+AK_{1}}{1+\left(1-A\right)K_{1}}$$

K_1_, K_2_ and A are defined by Eqs. (12), (13), (14)

(12) $$K_{1}=e^{\frac{-\left({∆G}_{1H2O}-m_{1}\left[\text{GuHCl}\right]\right)}{\mathrm{RT}}}$$

(13) $$K_{2}=e^{\frac{-\left({∆G}_{2H2O}-m_{2}\left[\text{GuHCl}\right]\right)}{\mathrm{RT}}}$$

(14) $$A=\frac{\left(Y_{i}-Y_{F}^{0}\right)}{\left(Y_{U}^{0}-Y_{F}^{0}\right)}$$

A, *m*_1_ and *m*_2_ were the fitted parameters, while Δ*G*°_1H2O_ and Δ*G*°_2H2O_ are the linearly extrapolated free energy differences at 0 M GuHCl [68] F to I, and I to U, respectively. Subsequently, the concentrations corresponding to 50% unfolding of the protein to an intermediate state ([GuHCl]_50, I_) and to an unfolded state ([GuHCl]_50, U_) were calculated by Eqs. (15), (16) and compared:

(15) $${\left[\text{GuHCl}\right]}_{50,I}=\frac{∆G{{}^{\circ}}_{1H2O}}{m_{1}}$$

(16) $${\left[\text{GuHCl}\right]}_{50,U}=\frac{∆G{{}^{\circ}}_{2H2O}}{m_{2}}$$

The fraction unfolded, A, was also calculated and plotted as a function of [GuHCl] to facilitate comparison of the shape of the unfolding curves between the three isoforms.

### ThT fluorescence assay of ApoE self-assembly

ApoE isoforms at ~ 25 μM in 20 mM PB (pH 7.4) were incubated in the presence of ThT at 37 °C in the SpectraMax i3 plate reader. Aqueous ThT stock solution was prepared at a concentration of 3.14 mM, filtered through a 0.2-μm pore size and used in a 1:2.2 ratio. Ninety-six-well plates were sealed with an optically clear polyolefin film to avoid evaporation (StarSeal Advanced Polyolefin Film, Starlab). The number of reading per well was set to 6, PMT voltage was set to high and blank spectra of the buffer were subtracted to protein fluorescence scans. Excitation wavelength was 440 nm, and emission at 483 nm was monitored every 15 min, with 3-s low orbital shakes before readings. Fluorescence intensity at a given time point (*F*) for each isoform was adjusted by subtracting the minimum fluorescence intensity value (*F*_min_), and not to the initial fluorescence, to account for the increasing temperature effect on ThT (from room temperature to 37 °C).

Adjusted fluorescence was plotted against time, and averaged traces for each isoform were obtained on GraphPad Prism. A minimum of three different production batches per isoform were used.

### Negative-stain transmission electron microscopy (TEM)

Morphology of ApoE after 24-h incubation at 37 °C was assessed by negative stain TEM. A droplet of sample (4 μL) was placed on 400-mesh carbon-coated grids (Agar Scientific, Essex, UK) and incubated for 1 min. After blotting the excess solution, the grid was washed with 4 μL filtered Milli-Q water and blotted. It was then negatively stained with 4 μL filtered 0.5% uranyl acetate for 40 s and blotted with filter paper. Grids were left to air-dry for at least 5 min before storage. Grids were examined on a Jeol Jem1400-plus transmission electron microscope (Jeol, USA) operated at 80 kV fitted with a Gatan Orius SC100 camera (UK).

### Separation of fibril pellet from supernatant

To probe the secondary structure of ApoE4 fibrils, pellets were isolated from supernatant by ultra-centrifugation of assembled fibrils at 60,000 rpm in an OptimaTM MAX Ultracentrifuge (Beckman Coulter) using a Beckman TLA120.2 fixed-angle rotor for 45 min at 4 °C. Supernatant was removed and replace by fresh 20 mM PB buffer. CD spectra of the whole fraction, supernatant, pellet and sonicated pellet (5-s sonication) were acquired as described for CD. The data were plotted graphically using GraphPad Prism but without conversion to molar ellipticity. Data for whole fraction, pellet and supernatant for the same sample were compared directly to compare secondary structure and protein content for each fraction.

### X-ray fiber diffraction

ApoE4 (100 μM in PB, pH 7.4) was incubated for 24 h at 37 °C to form fibrils and then the sample was centrifuged for 40 min at 21,100 rpm at 4 °C using a benchtop centrifuge (Mikro 22R, Hettich). The resulting pellet was resuspended in 400 μL filtered milliQ water, and this was repeated to remove salts that could interfere with the diffraction pattern. Finally, the pellet was resuspended in 30 μL filtered milliQ water. Ten microliters of concentrated fiber sample was suspended between two wax-filled capillaries and incubated in a sealed Petri dish at 4°C to form a partially aligned fiber sample [69].

X-ray diffraction images were collected using a Rigaku rotating anode source (CuKα) and a Saturn CCD + detector. Partially aligned fibers were placed in the x-ray beam and exposed for 30 or 60 s at specimen to detector distances of 50 and 100 mm. Diffraction patterns were converted to TIFF format using imosflm [70] and analyzed using CLEARER [71].

## Appendix A Supplementary data

Supplementary material Image 1

## References

[1] Selkoe D.J. (1991). The molecular pathology of Alzheimer's disease. Neuron..

[2] Rall S.C., Weisgraber K.H., Mahley R.W. (1982). Human apolipoprotein E. The complete amino acid sequence. J. Biol. Chem..

[3] Uddin M.S., Kabir M.T., Al Mamun A., Abdel-Daim M.M., Barreto G.E., Ashraf G.M. (2019). APOE and Alzheimer's disease: evidence mounts that targeting APOE4 may Combat Alzheimer's pathogenesis. Mol. Neurobiol..

[4] Wu L., Zhao L. (2016). ApoE2 and Alzheimer's disease: time to take a closer look. Neural Regen. Res..

[5] Dupont-Wallois L., Soulie C., Sergeant N., Wavrant-de Wrieze N., Chartier-Harlin M.C., Delacourte A. (1997). ApoE synthesis in human neuroblastoma cells. Neurobiol. Dis..

[6] Boschert U., Merlo-Pich E., Higgins G., Roses A.D., Catsicas S. (1999). Apolipoprotein E expression by neurons surviving excitotoxic stress. Neurobiol. Dis..

[7] Utermann G. (1982). Apolipoprotein E (role in lipoprotein metabolism and pathophysiology of hyperlipoproteinemia type III). Ric. Clin. Lab..

[8] Mahley R.W., Innerarity T.L., Rall S.C., Weisgraber K.H. (1984). Plasma lipoproteins: apolipoprotein structure and function. J. Lipid Res..

[9] Hauser P.S., Narayanaswami V., Ryan R.O. (2011). Apolipoprotein E: from lipid transport to neurobiology. Prog. Lipid Res..

[10] Frieden C., Wang H., Ho C.M.W. (2017). A mechanism for lipid binding to apoE and the role of intrinsically disordered regions coupled to domain-domain interactions. Proc. Natl. Acad. Sci. U. S. A..

[11] Mahley R.W., Huang Y. (2012). Small-molecule structure correctors target abnormal protein structure and function: structure corrector rescue of apolipoprotein E4-associated neuropathology. J. Med. Chem..

[12] Winkler K., Scharnagl H., Tisljar U., Hoschutzky H., Friedrich I., Hoffmann M.M. (1999). Competition of Abeta amyloid peptide and apolipoprotein E for receptor-mediated endocytosis. J. Lipid Res..

[13] Namba Y., Tomonaga M., Kawasaki H., Otomo E., Ikeda K. (1991). Apolipoprotein E immunoreactivity in cerebral amyloid deposits and neurofibrillary tangles in Alzheimer's disease and kuru plaque amyloid in Creutzfeldt–Jakob disease. Brain Res..

[14] Rebeck G.W., Reiter J.S., Strickland D.K., Hyman B.T. (1993). Apolipoprotein E in sporadic Alzheimer's disease: allelic variation and receptor interactions. Neuron..

[15] Schmechel D., Saunders A., Strittmatter W., Grain B., Hulette C., Jou S. (1993). Increased amyloid b-peptide deposition in cerebral cortex as a consequenceof apolipoprotein E phenotype in late onset Alzheimer Disease. PNAS USA..

[16] Tiraboschi P., Hansen L.A., Masliah E., Alford M., Thal L.J., Corey-Bloom J. (2004). Impact of APOE genotype on neuropathologic and neurochemical markers of Alzheimer disease. Neurology..

[17] Reiman E.M., Chen K., Liu X., Bandy D., Yu M., Lee W. (2009). Fibrillar amyloid-beta burden in cognitively normal people at 3 levels of genetic risk for Alzheimer's disease. Proc. Natl. Acad. Sci. U. S. A..

[18] Morris J.C., Roe C.M., Xiong C., Fagan A.M., Goate A.M., Holtzman D.M. (2010). APOE predicts amyloid-beta but not tau Alzheimer pathology in cognitively normal aging. Ann. Neurol..

[19] Deane R., Sagare A., Hamm K., Parisi M., Lane S., Finn M.B. (2008). apoE isoform-specific disruption of amyloid beta peptide clearance from mouse brain. J. Clin. Invest..

[20] Liao F., Li A., Xiong M., Bien-Ly N., Jiang H., Zhang Y. (2018). Targeting of nonlipidated, aggregated apoE with antibodies inhibits amyloid accumulation. J. Clin. Invest..

[21] Shi Y., Yamada K., Liddelow S.A., Smith S.T., Zhao L., Luo W. (2017). ApoE4 markedly exacerbates tau-mediated neurodegeneration in a mouse model of tauopathy. Nature..

[22] Wang C., Najm R., Xu Q., Jeong D.E., Walker D., Balestra M.E. (2018). Gain of toxic apolipoprotein E4 effects in human iPSC-derived neurons is ameliorated by a small-molecule structure corrector. Nat. Med..

[23] Chen J., Li Q., Wang J. (2011). Topology of human apolipoprotein E3 uniquely regulates its diverse biological functions. Proc. Natl. Acad. Sci. U. S. A..

[24] Dong L.M., Parkin S., Trakhanov S.D., Rupp B., Simmons T., Arnold K.S. (1996). Novel mechanism for defective receptor binding of apolipoprotein E2 in type III hyperlipoproteinemia. Nat. Struct. Biol..

[25] Dong J., Peters-Libeu C.A., Weisgraber K.H., Segelke B.W., Rupp B., Capila I. (2001). Interaction of the N-terminal domain of apolipoprotein E4 with heparin. Biochemistry..

[26] Morrow J.A., Hatters D.M., Lu B., Hochtl P., Oberg K.A., Rupp B. (2002). Apolipoprotein E4 forms a molten globule. A potential basis for its association with disease. J. Biol. Chem..

[27] Mahley R.W., Weisgraber K.H., Huang Y. (2006). Apolipoprotein E4: a causative factor and therapeutic target in neuropathology, including Alzheimer's disease. Proc. Natl. Acad. Sci. U. S. A..

[28] Mahley R.W., Huang Y. (2012). Apolipoprotein e sets the stage: response to injury triggers neuropathology. Neuron..

[29] Hatters D.M., Zhong N., Rutenber E., Weisgraber K.H. (2006). Amino-terminal domain stability mediates apolipoprotein E aggregation into neurotoxic fibrils. J. Mol. Biol..

[30] Kraft L., Serpell L.C., Atack J.R. (2019). A biophysical approach to the identification of novel ApoE chemical probes. Biomolecules..

[31] Erickson H.P. (2009). Size and shape of protein molecules at the nanometer level determined by sedimentation, gel filtration, and electron microscopy. Biol. Proced. Online.

[32] Provencher S.W., Glockner J. (1981). Estimation of globular protein secondary structure from circular dichroism. Biochemistry..

[33] van Stokkum I.H., Spoelder H.J., Bloemendal M., van Grondelle R., Groen F.C. (1990). Estimation of protein secondary structure and error analysis from circular dichroism spectra. Anal. Biochem..

[34] Lobley A., Whitmore L., Wallace B.A. (2002). DICHROWEB: an interactive website for the analysis of protein secondary structure from circular dichroism spectra. Bioinformatics..

[35] Whitmore L., Wallace B.A. (2004). DICHROWEB, an online server for protein secondary structure analyses from circular dichroism spectroscopic data. Nucleic Acids Res..

[36] Whitmore L., Wallace B.A. (2008). Protein secondary structure analyses from circular dichroism spectroscopy: methods and reference databases. Biopolymers..

[37] Sreerama N., Woody R.W. (2000). Estimation of protein secondary structure from circular dichroism spectra: comparison of CONTIN, SELCON, and CDSSTR methods with an expanded reference set. Anal. Biochem..

[38] LeVine H. (1999). Quantification of beta-sheet amyloid fibril structures with thioflavin T. Methods Enzymol..

[39] Chen L., Morris K., Laybourn A., Elias D., Hicks M.R., Rodger A. (2010). Self-assembly mechanism for a naphthalene-dipeptide leading to hydrogelation. Langmuir.

[40] Tayeb-Fligelman E., Tabachnikov O., Moshe A., Goldshmidt-Tran O., Sawaya M.R., Coquelle N. (2017). The cytotoxic *Staphylococcus aureus* PSMalpha3 reveals a cross-alpha amyloid-like fibril. Science..

[41] Morris K.L., Rodger A., Hicks M.R., Debulpaep M., Schymkowitz J., Rousseau F. (2013). Exploring the sequence-structure relationship for amyloid peptides. Biochem. J..

[42] Serpell L. (2000). Alzheimer's amyloid fibrils: structure and assembly. Biochim. Biophys. Acta.

[43] Goldsbury C., Kistler J., Aebi U., Arvinte T., Cooper G. (1999). Watching amyloid fibrils grow by time-lapse atomic force microscopy. J. Mol. Biol..

[44] Jahn T.R., Makin O.S., Morris K.L., Marshall K.E., Tian P., Sikorski P. (2010). The common architecture of cross-beta amyloid. J. Mol. Biol..

[45] Kanekiyo T., Xu H., Bu G. (2014). ApoE and Abeta in Alzheimer's disease: accidental encounters or partners?. Neuron..

[46] Bu G. (2009). Apolipoprotein E and its receptors in Alzheimer's disease: pathways, pathogenesis and therapy. Nat. Rev. Neurosci..

[47] Hatters D.M., Budamagunta M.S., Voss J.C., Weisgraber K.H. (2005). Modulation of apolipoprotein E structure by domain interaction: differences in lipid-bound and lipid-free forms. J. Biol. Chem..

[48] Clement-Collin V., Barbier A., Dergunov A.D., Visvikis A., Siest G., Desmadril M. (2006). The structure of human apolipoprotein E2, E3 and E4 in solution. 2. Multidomain organization correlates with the stability of apoE structure. Biophys. Chem..

[49] Huang Y., Mahley R.W. (2014). Apolipoprotein E: structure and function in lipid metabolism, neurobiology, and Alzheimer's diseases. Neurobiol. Dis..

[50] Argyri L., Skamnaki V., Stratikos E., Chroni A. (2011). A simple approach for human recombinant apolipoprotein E4 expression and purification. Protein Expr. Purif..

[51] Barbier A., Clement-Collin V., Dergunov A.D., Visvikis A., Siest G., Aggerbeck L.P. (2006). The structure of human apolipoprotein E2, E3 and E4 in solution 1. Tertiary and quaternary structure. Biophys. Chem..

[52] Acharya P., Segall M.L., Zaiou M., Morrow J., Weisgraber K.H., Phillips M.C. (2002). Comparison of the stabilities and unfolding pathways of human apolipoprotein E isoforms by differential scanning calorimetry and circular dichroism. Biochim. Biophys. Acta.

[53] Wetterau J.R., Aggerbeck L.P., Rall S.C., Weisgraber K.H. (1988). Human apolipoprotein E3 in aqueous solution. I. Evidence for two structural domains. J. Biol. Chem..

[54] Wolfe L.S., Calabrese M.F., Nath A., Blaho D.V., Miranker A.D., Xiong Y. (2010). Protein-induced photophysical changes to the amyloid indicator dye thioflavin T. Proc. Natl. Acad. Sci. U. S. A..

[55] Biancalana M., Makabe K., Koide A., Koide S. (2009). Molecular mechanism of thioflavin-T binding to the surface of beta-rich peptide self-assemblies. J. Mol. Biol..

[56] Salinas N., Colletier J.P., Moshe A., Landau M. (2018). Extreme amyloid polymorphism in *Staphylococcus aureus* virulent PSMalpha peptides. Nat. Commun..

[57] Glenner G., Eanes E., Bladen H., Linke R., Termine J. (1974). b-Pleated sheet fibrils. A comparison of native amyloid with synthetic protein fibrils. J. Histochem. Cytochem..

[58] Sipe J.D., Benson M.D., Buxbaum J.N., Ikeda S., Merlini G., Saraiva M.J. (2014). Nomenclature 2014: amyloid fibril proteins and clinical classification of the amyloidosis. Amyloid.

[59] Serpell L., Smith J. (2000). Direct visualisation of the beta-sheet structure of synthetic Alzheimer's amyloid. J. Mol. Biol..

[60] Pauling L., Corey R.B. (1951). Configuration of polypeptide chains. Nature..

[61] Astbury W.T., Woods H.J., Bragg W.L. (1934). X-ray studies of the structure of hair, wool, and related fibres. II. The molecular structure and elastic properties of hair keratin. Philos. Trans. R. Soc. Lond. A.

[62] Marshall K.E., Vadukul D.M., Dahal L., Theisen A., Fowler M.W., Al-Hilaly Y. (2016). A critical role for the self-assembly of Amyloid-beta1-42 in neurodegeneration. Sci. Rep..

[63] Belorgey D., Irving J.A., Ekeowa U.I., Freeke J., Roussel B.D., Miranda E. (2011). Characterisation of serpin polymers in vitro and in vivo. Methods..

[64] Pericak-Vance M., Bebout J., Gaskell P., Hung W.-Y., Alberts M., Walker A. (1991). Linkage studies in familial Alzheimer's disease: evidence for chromosome 19 linkage. Am. J. Hum. Genet..

[65] Schuck P. (2000). Size-distribution analysis of macromolecules by sedimentation velocity ultracentrifugation and Lamm equation modeling. Biophys. J..

[66] Laue T.M., Shah B.D., Ridgeway T.M., Pelletier S.L. (1992). Computer-aided interpretation of analytical sedimentation data for proteins. Analytical Ultracentrifugation in Biochemistry and Polymer Science.

[67] Kishore D., Kundu S., Kayastha A.M. (2012). Thermal, chemical and pH induced denaturation of a multimeric beta-galactosidase reveals multiple unfolding pathways. PLoS One.

[68] Tendian S.W., Myszka D.G., Sweet R.W., Chaiken I.M., Brouillette C.G. (1995). Interdomain communication of T-cell CD4 studied by absorbance and fluorescence difference spectroscopy measurements of urea-induced unfolding. Biochemistry..

[69] Morris K.L., Serpell L.C. (2012). X-ray fibre diffraction studies of amyloid fibrils. Methods Mol. Biol..

[70] CCP4 (1994). The CCP4 suite programs for crystallography. Acta Cryst.

[71] Makin O.S., Sikorski P., Serpell L.C. (2007). CLEARER: a new tool for the analysis of x-ray fibre diffraction patterns and diffraction simulation from atomic structural models. Appl. Cryst..

