# Supplementary material for: The Molecular Basis for Apolipoprotein E4 as the Major Risk Factor for Late-Onset Alzheimer's Disease
Source: J Mol Biol. 2019 May 31;431(12):2248–65. doi: 10.1016/j.jmb.2019.04.019 (PMC6556554; doi:10.1016/j.jmb.2019.04.019)
Supplement: Supplementary file 1 — Supplementary material [file mmc1.docx]

**Supplementary information: The molecular basis of the major risk factor for Alzheimer’s disease: structural studies of ApoE isoforms**

**Ana-Caroline Raulin^1^*, Lucas Kraft^2^*, Youssra K. Al-Hilaly^1^, John E. McGeehan^3^, John R. Atack^2a^, Louise Serpell^1x^**

**Email addresses:**

**Ana-Caroline Raulin** [**A.Raulin@sussex.ac.uk**](mailto:A.Raulin@sussex.ac.uk)**;**

**Lucas Kraft** [**L.V.Kraft@sussex.ac.uk**](mailto:L.V.Kraft@sussex.ac.uk)

**Youssra Al-Hilaly** [**ya45@sussex.ac.uk**](mailto:ya45@sussex.ac.uk)

**John McGeehan John.McGeehan@port.ac.uk**

**John Atack** [**j.Atack@sussex.ac.uk**](mailto:j.Atack@sussex.ac.uk)

**Louise Serpell L.C.Serpell@sussex.ac.uk**

**^a^ Current address: Medicines Discovery Institute, Cardiff University, Cardiff, CF10 3AT, UK**

**x corresponding author**

**Supplementary Materials and methods**

**Mutagenesis, expression and purification of ApoE isoforms**

The codon-optimized ApoE4 gene was synthesized and cloned into a pET17b vector at the NdeI/HindIII site by Thermo Fisher Scientific (pET17b_ApoE4). A six-histidine tag, thioredoxin (TRX) and HRV 3C protease cleavage site are found upstream of the ApoE4 gene. The plasmid containing HRV 3C protease gene fused with glutathione S-transferase (GST) was provided by Dr Antony Oliver (Genome Damage and Stability Centre, University of Sussex).

The ApoE2 and ApoE3 full-length variants were created by site directed mutagenesis (QuikChange Lightening site directed mutagenesis kit, Agilent) using the pET17b_ApoE4 plasmid as template and primers 5'-ggtgcagatatggaagatgtttgtggtcgtctgg-3' and 5’-gccgatgatctgcagaaatgtctggcagtttatcag-3’ respectively.

Recombinant ApoE proteins were expressed in *E. coli* Rosetta2(DE3) cells (#71400-3, Merck Millipore) in Luria-Bertani (LB) broth supplemented with 100 µg/mL ampicillin. Protein expression was induced in bacteria grown to an optical density of 600 nm (OD_600_) of 0.7-0.9 with 1 mM isopropyl β-D-1-thiogalactopyranoside (IPTG) for 2 h at 37°C. Bacteria were then pelleted and resuspended in ice-cold lysis buffer (50 mM HEPES, 240 mM NaCl, 5 mM MgCl2, 10 mM imidazole, 1 mM DTT, 10% (v/v) glycerol, 0.05 % (v/v) Tween-20, 7 U/mL DNAse I, 1 protease inhibitor tablet per 50 mL, pH 8.0). Cell lysis was achieved by sonication and cell debris separated by centrifugation. Proteins were affinity-purified with Talon® beads. After equilibration of the Talon® beads with the soluble component of the cell lysate, the beads were washed with talon binding buffer (50 mM HEPES, 240 mM NaCl, 20 mM imidazole, 10% (v/v) glycerol, 0.05% (v/v) Tween-20, pH 8.0) and protein eluted with talon elution buffer (50 mM HEPES, 240 mM NaCl, 300 mM imidazole, 10% (v/v) glycerol, 0.05% (v/v) Tween-20, pH 8.0). The elution fractions were then applied on a HiTrap heparin column (GE Healthcare) and extensively washed with either size exclusion buffer for ApoE3 and ApoE4 (20 mM HEPES, 300 mM NaCl, 10% (v/v) glycerol, pH 8.0) or heparin binding buffer for ApoE2 (20 mM HEPES, 240 mM NaCl, 10% (v/v) glycerol, pH 8.0). After on-column digestion with HRV 3C protease overnight at 4°C, the cleaved TRX tag was eluted with size exclusion or heparin binding buffer and ApoE proteins then eluted by applying a linear salt gradient. Elution fractions were pooled, applied on a HiLoad Superdex 26/600 pg 200 column (GE Healthcare) and all proteins eluted in size exclusion chromatography (SEC) buffer (20 mM HEPES, 300 mM NaCl, 10% (v/v) glycerol, pH 8.0). ApoE-containing fractions were concentrated by centrifugation using VivaSpin 20 concentrators (Sartorius, MWCO 5000 Da) and protein stored at -80°C. All steps and purity of samples were analysed by sodium dodecyl sulfate polyacrylamide gel electrophoresis (SDS PAGE).

**Expression and purification of HRV 3C protease**

The GST-HRV 3C protease fusion protein was expressed in *E. coli* Rosetta2(DE3) cells in Luria-Bertani (LB) broth supplemented with 100 µg/mL ampicillin and 35 µg/mL chloramphenicol. Protein expression was induced in bacteria grown to an OD_600_ of 0.6 with 0.4 mM IPTG overnight at 20°C. Cells were pelleted and re-suspended in ice-cold lysis buffer (50 mM HEPES pH 8.0, 1000 mM NaCl, 5 mM MgCl_2_, 1 mM DTT, 7 U/mL DNAse I, 1 protease inhibitor tablet per 50 mL) and cell lysis was achieved by sonication. After separation of insoluble components by centrifugation the protease was affinity purified using a GSTrap FF column (GE Healthcare). After passing the soluble lysis fractions through the column, the column was extensively washed with binding buffer (50 mM HEPES pH 8.0, 1000 mM NaCl, 1 mM EDTA, 1 mM DTT) and 3C protease then eluted with 10 mM reduced glutathione. Protease-containing fractions were pooled, concentrated as described above, re-buffered into storage buffer (50 mM HEPES pH 8.0, 1000 mM NaCl, 1 mM EDTA, 1 mM DTT, 20% (v/v) glycerol) and stored at -80˚C.

**Supplementary Results**

**
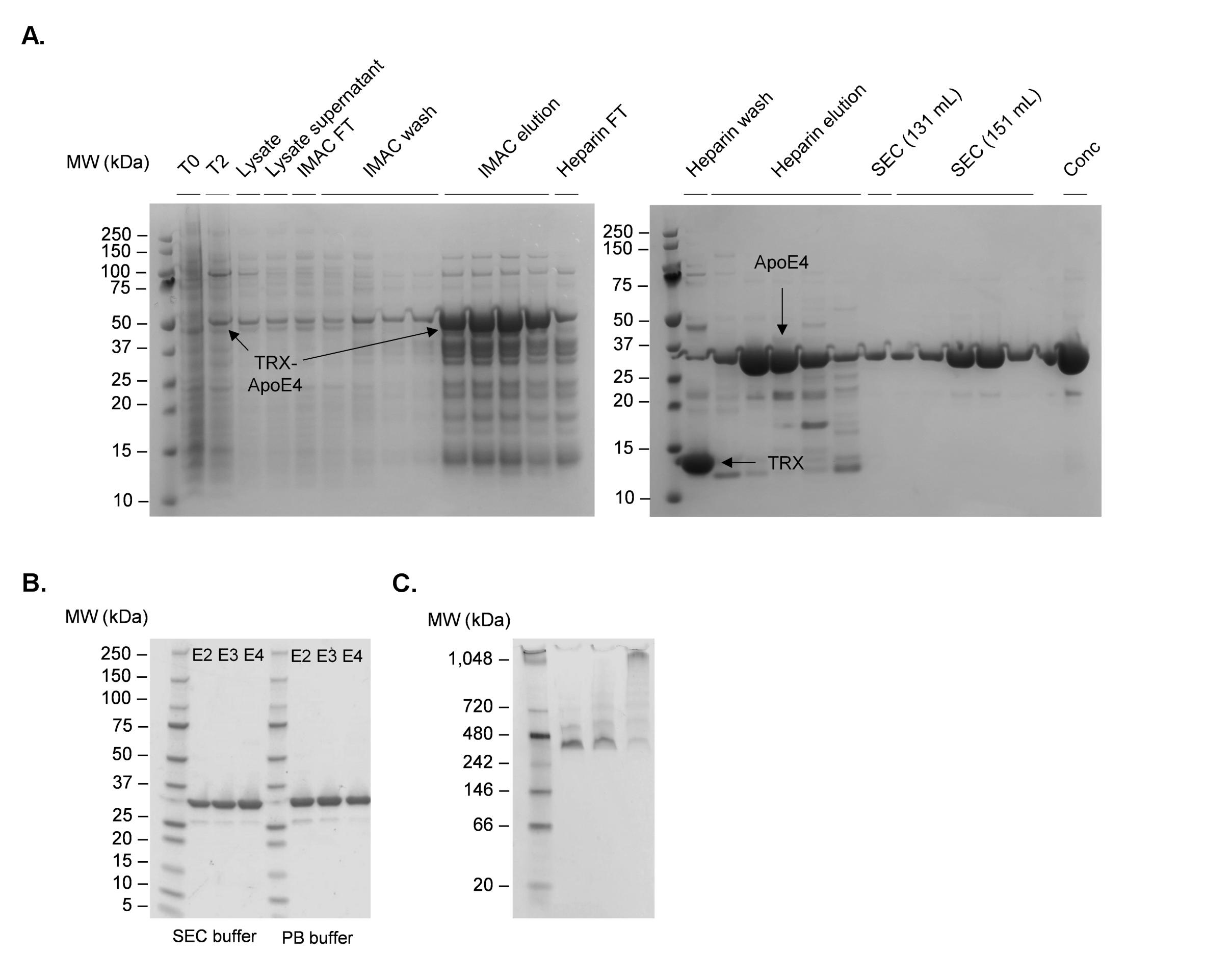
**

**Figure S1. Purification of ApoE4 and gel electrophoresis**

A. Example gels of the purification of ApoE4 are shown. The thioredoxin (TRX) ApoE4 fusion protein (TRX-ApoE4) is highly expressed in Rosetta2(DE3) cells (compare T0 versus T2) and shows good solubility in the lysis buffer used; soluble protein is found in the lysate supernatant as demonstrated on the left gel. A primary capture step by immobilized metal ion affinity chromatography (IMAC) enriches the protein of interest and removes major impurities. However, fusion protein is found in the flow through (IMAC FT), as well as wash fractions (IMAC wash) of the IMAC. Nevertheless, most of the TRX-ApoE4 fusion protein is found in the elution fractions (IMAC elution). An intermediate purification step takes advantage of the heparin binding sites in ApoE4. The TRX-ApoE4 fusion protein shows excellent heparin binding affinity as only minimal amount of protein is seen in the flow through of the column (Heparin FT). Cleavage of the TRX solubility tag by HRV 3C protease was performed on the heparin column. The right gel demonstrates successful digestion of the solubility tag; pleasingly, TRX protein elutes at lower salt concentrations and is therefore found in the wash fractions (Heparin wash). ApoE4 on the other hand elutes at higher salt concentrations (Heparin elution). A final purification step by size exclusion chromatography (SEC) eliminates last impurities and aggregates that may have formed. ApoE4 elutes at two apparent elution volumes. One smaller fraction at 131 mL and the major fraction at 151 mL. This suggests that different oligomeric species are present. Only the major fractions at 151 mL were pooled and concentrated. The final concentrate (Conc.) has high purity, however, a minor impurity at 22 kDa is seen most likely due to minor fragmentation.

B. SDS-PAGE showing that all three ApoE isoforms migrated to ~34 kDa in SEC buffer and after dialysis into 20 mM PB, pH 7.4.

C. Native PAGE of ApoE isoforms in 20 mM PB showing a common band at >480 kDa, and the presence of higher multimeric species in ApoE4 (between 480 and 720 kDa).

**Figure S2. In-line SEC-SAXS.**

Scattering profiles corresponding to curves A. 296-302 (ApoE2), B. 296-303 (ApoE3) and C. 295-302 (ApoE4) were selected for analysis and averaging. The integral of ratio to background (or scattering intensity) and the radius of gyration R_G_ is plotted against the frame number.

**
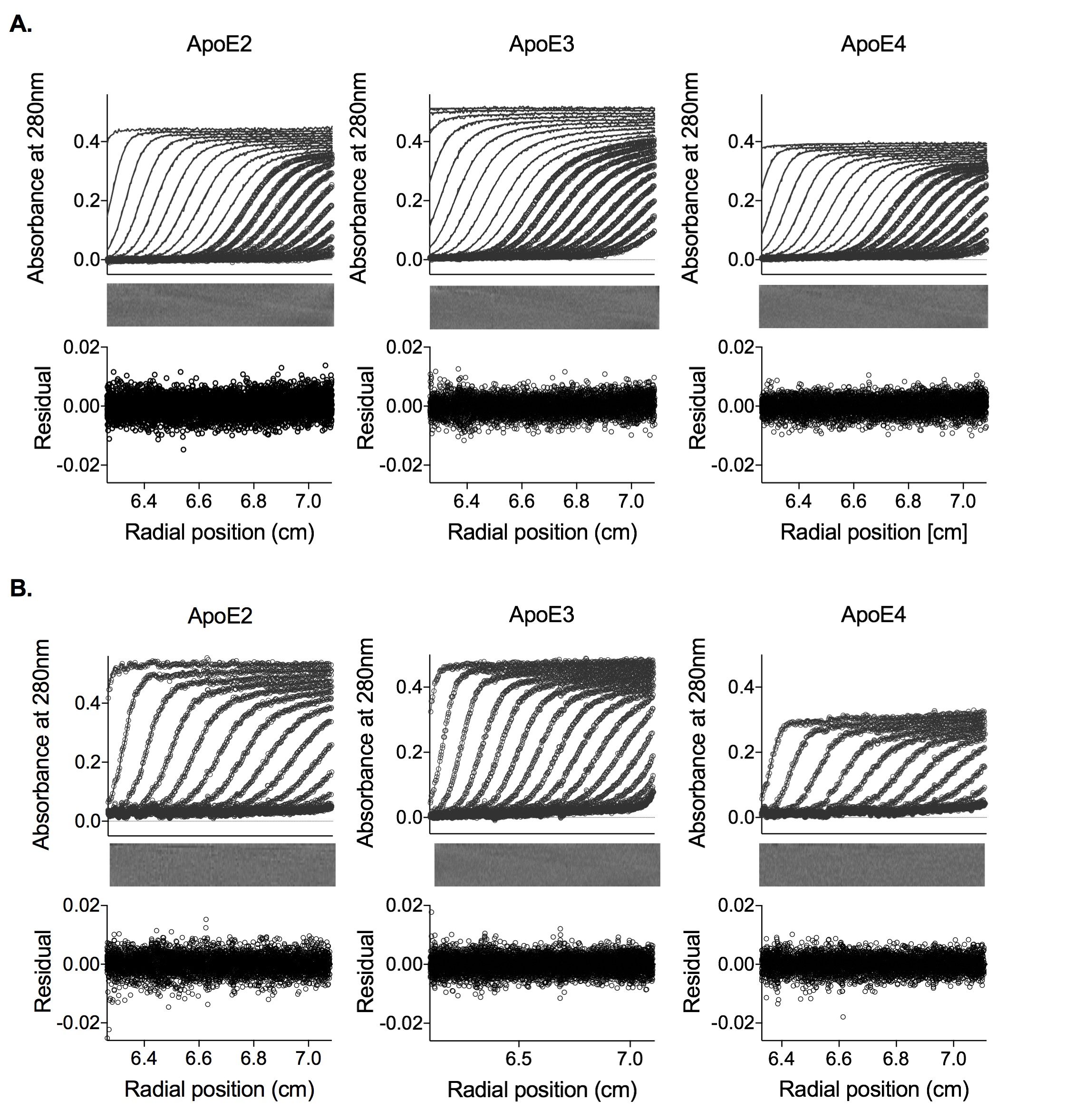
**

**Figure S3. Hydrodynamic behaviour of recombinant ApoE isoforms**

Sedimentation velocity profiles of ApoE2, ApoE3 and ApoE4 (8 μM) in A. size exclusion buffer and B. in 20 mM PB, pH 7.4 were recorded using an analytical ultracentrifuge at 20°C, at a speed of 40,000 rpm and A280nm was scanned every 25 min at radial intervals of 0.003 cm. Curves were fitted to the Lamm equation with the SEDFIT program (upper panels). Greyscale of evenly distributed bitmap (medium panels) indicates no systematic errors in the fit, and residuals (bottom panels) plotted as a function of radial position attest of the quality of the fit.

**
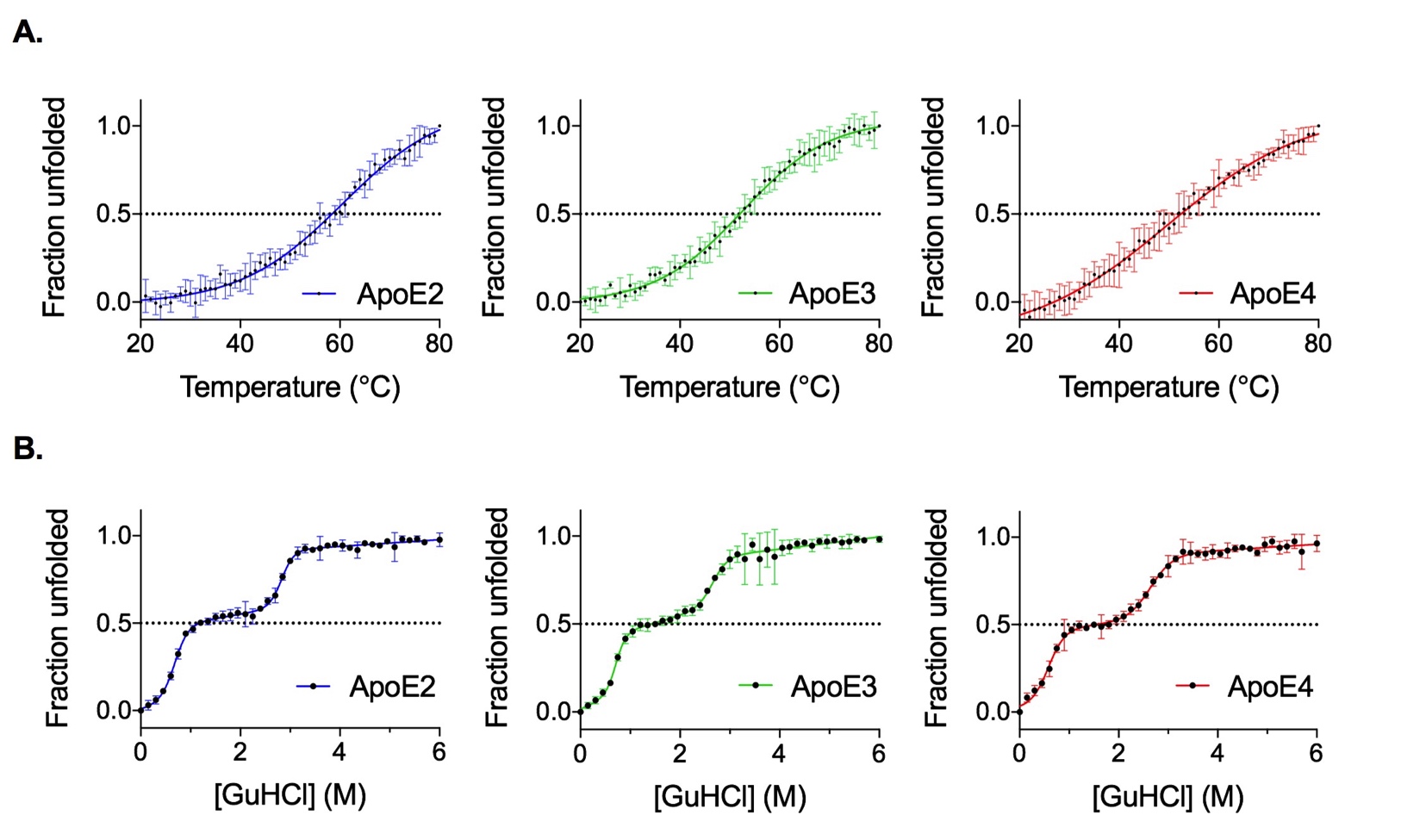
**

**Figure S4. Fitting of ApoE isoforms denaturation curves**

A. Thermal denaturation curves as fraction unfolded with experimental data points (closed circles) and error bars depicting the SD of three individual experiments (different production batches).

B. Chemical denaturation curves as fraction unfolded with experimental data points (closed circles) and error bars depicting the SD of three individual experiments (different production batches).

**
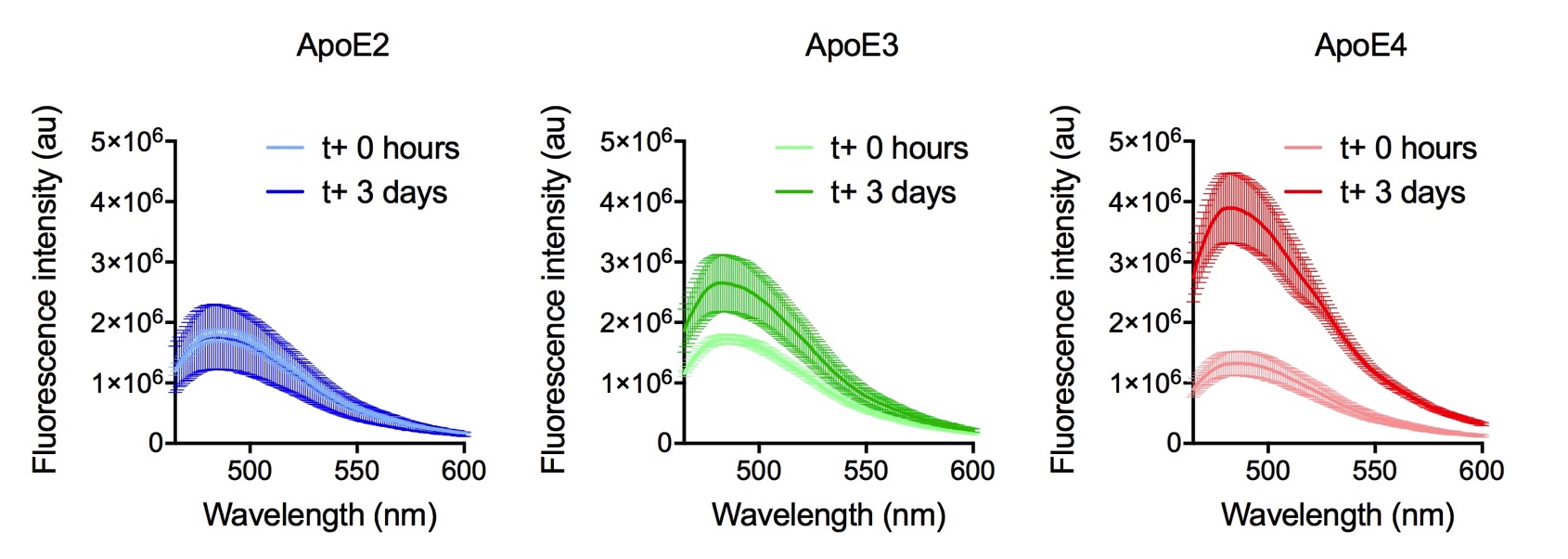
**

**Figure S5. ThT fluorescence scan**

Comparison between ThT fluorescence scans taken before and after a 3-day incubation period shows significant differences in signal only for ApoE4, indicative of self-assembly.
